# Impact of comorbidities on people with and without cancer early in the COVID‐19 pandemic: An observational study

**DOI:** 10.1002/cam4.6212

**Published:** 2023-06-03

**Authors:** Carolyn E. Schwartz, Bruce D. Rapkin

**Affiliations:** ^1^ DeltaQuest Foundation, Inc. Concord Massachusetts USA; ^2^ Department of Medicine Tufts University Medical School Boston Massachusetts USA; ^3^ Department of Orthopaedic Surgery Tufts University Medical School Boston Massachusetts USA; ^4^ Department of Epidemiology and Population Health Albert Einstein College of Medicine Bronx New York USA

**Keywords:** cancer survivor, comparison, COVID, quality of life, social determinants of health

## Abstract

**Background:**

The present study sought to investigate how comorbidity burden influences cancer survivors' quality of life (QoL) and the challenges/adaptations during the coronavirus disease 2019 (COVID) pandemic, and to examine how appraisal processes are related to this impact.

**Methods:**

This cross‐sectional study, administered in spring/summer 2020, compared cancer survivors to a general‐population comparison sample. QoL was assessed with standardized tools. COVID‐specific questions included selected items compiled by the US National Institutes of Health, and cognitive appraisal processes were assessed using the QoL Appraisal Profile_v2_ Short‐Form. Principal components analysis reduced the number of comparisons. Multivariate analysis of covariance investigated group differences in QoL, COVID‐specific variables, and cognitive‐appraisal processes. Linear regression investigated group differences in COVID‐specific variables as a function of cognitive‐appraisal processes, QoL, demographic covariates, and their interactions.

**Results:**

Cancer survivors fared substantially better than non‐cancer participants in QoL and cognitive functioning when they had no other comorbidities, but substantially worse on QoL when they had three or more comorbidities. Cancer survivors with no comorbidities were less likely to feel worried about COVID, less likely to engage in self‐protection, and prioritized engaging in problem‐focused and prosocial actions compared to non‐cancer participants. Conversely, cancer survivors confronted with multiple comorbidities exhibited more proactive self‐protection and experienced more anxiety about the pandemic.

**Conclusion:**

The impact of having multiple comorbidities in the context of cancer is associated with notable differences in social determinants of health, QoL outcomes, COVID‐specific challenges/adaptations, and appraisal of QoL. These findings provide an empirical basis for implementing appraisal‐based coping interventions.

## Abstract 2

This cross‐sectional study, administered in Spring/Summer 2020, compared cancer survivors (*n* = 520) to a general‐population comparison sample (*n* = 441). The impact of having multiple comorbidities in the context of cancer is associated with notable differences in social determinants of health, quality of life (QOL) outcomes, COVID‐specific challenges/adaptations, and appraisal of QOL.

## 1 INTRODUCTION

The coronavirus disease 2019 (COVID) has impacted people the world over. Early in the pandemic, it became apparent that COVID magnified other health risk factors, including immune‐compromise and multiple morbidities.^1^ It also magnified the impact of social determinants of health, such as financial strain, minority‐group membership, and sociodemographic indicators.^2^, ^3^, ^4^ Despite the admirable progress made in the development of vaccines^5^ and treatments^6^ that reduce the health consequences of COVID, the quality of life (QoL) impact of COVID continues.^7^ This is especially true for people affected by cancer. For the purposes of the present work, “cancer survivor” refers to someone who has ever received a diagnosis of cancer, even if still receiving treatment.

While many medical illnesses can make one more vulnerable to the effects of COVID,^8^ the conditions collectively known as “cancer” have posed particular challenges during the pandemic.^9^ Impaired immunity, either related to cancer etiology or treatment effects, increase COVID risk and reduce the efficacy of vaccines.^10^ Risk factors for cancer such as smoking and obesity are associated with many other chronic health conditions, particularly diseases that are most prevalent in later life.^11^, ^12^ Accordingly, people with a history of cancer (cancer survivors) and other comorbid conditions may be particularly vulnerable to social and environmental circumstances that tax their capacities to cope and to maintain QoL.^13^

A growing literature suggests that the negative impact of the COVID pandemic spans multiple life domains,^14^ including economic hardship; interpersonal conflict, risk‐taking behaviors, and emotional distress.^15^ Positive impacts have also emerged, such as social support, self‐protective behaviors, and salutogenic coping, such as prosocial behaviors and attitudinal perspectives that transform negatives into hidden positives.^16^, ^17^ This “yin” and “yang” of the repercussions of COVID mean that even in the context of a life‐changing worldwide pandemic, how one copes and the choices one makes differentiate those who thrive from those who do not. Understanding personal factors that have influenced adaptation during COVID could guide the development of interventions to help individuals living with multiple chronic conditions cope with challenges due to COVID and future pandemics.

Characterizing how people think about QoL is key to understanding individual differences in the impact of illness.^18^, ^19^ A substantial evidence base supports the importance of cognitive appraisal in explaining variance in QoL across patient populations, after medical interventions, over time, and across types of stressors.^20^, ^21^ QoL appraisal processes reflect one's frame of reference, recall of relevant events, evaluative comparisons, and the relative importance of different experiences in judging one's own QoL.^21^, ^22^ It follows that people dealing with different life circumstances and health challenges might experience a crisis like the COVID pandemic in markedly different ways. As such it is important to examine appraisal processes related to better and worse adaptation to COVID among diverse cancer survivors.

The present study sought to investigate how burdens of cancer and comorbid illness combine to influence QoL and challenges/adaptations associated with COVID, and to examine how appraisal processes are related to this impact, by comparing people with cancer to a general‐population comparison sample.

## 2 METHODS

### 2.1 Design

This cross‐sectional study was administered in spring/summer 2020 as part of a longitudinal study.

### 2.2 Sample and procedure

Cancer survivors were recruited from Rare Patient Voice/What Next cancer panels, for which cancer diagnosis was vetted based on attendance at cancer‐related patient‐advocacy conferences. We included all cancer diagnoses. Information on cancer stage was not available. The non‐cancer group was recruited from the IPSOS‐Insight general‐population subsample to be nationally representative in terms of age distribution, gender, region, and income. Number of non‐cancer comorbidities for both groups was based on respondent self‐report. Anyone in the IPSOS panel who reported cancer as a comorbidity was excluded from analysis. Participants were not paid for their participation, although Ipsos Insight used its usual respondent point‐related incentives.

Eligible participants were age 18 or older and able to complete an online questionnaire. Participants with motor, visual, and/or other problems that made it difficult for them to complete the web‐based survey enlisted the assistance of someone else to enter the participant's answers. This survey was administered through the secure Alchemer engine (www.alchemer.com), which is compliant with the US Health Insurance Portability and Accountability Act. The protocol was reviewed and approved by the New England Independent Review Board (NEIRB #2021164), and all participants provided informed consent prior to beginning the survey.

### 2.3 Measures

#### 2.3.1 Quality of life ( *QoL* )

QoL was assessed with standardized tools appropriate for use across all populations. The PROMIS‐10 is a brief measure of general physical and mental health over the past 7 days,^23^ with higher scores reflecting better function. The Neuro‐QoL Applied Cognition^24^ assessed perceived difficulties in everyday cognitive abilities (memory, attention, and decision‐making) and in applications of mental function (planning, organizing, calculating, working with memory, and learning) over the past 7 days, with higher scores reflecting worse cognitive function. Well‐being was assessed using the Neuro‐QoL Positive Affect and Well‐being short‐form, with a more general recall period of “lately”,^24^ the Ryff Psychological Well‐being scale subscales for environmental mastery and purpose in life with a general time period that is not specified,^25^, ^26^ and the DeltaQuest Wellness Measure© (DQ Wellness), which taps into attitudes, perspectives, and behaviors relevant to wellness over the past week.^27^ For the well‐being measures, higher scores reflect better well‐being.

#### 2.3.2 COVID‐specific questions

COVID‐specific questions included selected items compiled by the US National Institutes of Health (NIH) Office of Behavioral and Social Sciences Research and the NIH Disaster Research program^28^ (see Table S1 for full detail, including recall period for items). These items assessed: infection status; self‐protection (e.g., hand‐washing, social distancing, wearing mask); risk‐taking (e.g., going to public places such as a grocery store, restaurant, or social gathering); hardship (i.e., current economic problems within the individual/family; worries about future economic concerns of the individual/family; inadequate access to healthcare; and housing instability); interpersonal conflict (i.e., anger and conflict with others in one's environment); emotional distress about the pandemic (i.e., dysphoric rumination); post‐traumatic growth (i.e., renewed appreciation for sources of personal meaning and value); altruism (i.e., COVID‐specific generosity to others such as bringing food or medicine, helping with childcare, etc.); coping with lockdown (i.e., health‐enhancing behavioral strategies); and social support (i.e., sources of emotional support). The post‐traumatic growth scale used four items adapted with permission from the Post‐traumatic Growth Inventory.^29^, ^30^, ^31^

#### 2.3.3 Cognitive appraisal processes

Cognitive appraisal processes were assessed using the QoL Appraisal Profile_v2_ Short‐Form. This short form contains items assessing patterns of emphasis (i.e., combinatory algorithm; 9 items), standards of comparison (9 items), sampling of experience (4 items), and goal delineation.^32^ This measure has been used to investigate the cognitive appraisal processes underlying QoL assessment,^32^, ^33^, ^34^ and to capture response‐shift effects in longitudinal data.^35^, ^36^

#### 2.3.4 Demographic characteristics

*Demographic characteristics* included age, gender, years since diagnosis,* US state‐based region, race, education, difficulty paying bills, cohabitation/marital status, whether the person lives alone, height and weight (to compute body mass index), smoking status, comorbidities, and whether the individual had been infected with Sars Cov‐2.

### 2.4 Statistical analysis

Descriptive statistics summarized the sample demographic characteristics. Analysis of variance (ANOVA) was used to compare demographic characteristics for cancer and non‐cancer participants (group). Chi‐squared tests compared comorbidities by group, and Pearson correlations summarized associations between appraisal and QoL, COVID‐specific variables, and demographics, separately by group. To reduce the number of statistical comparisons, principal components analysis (PCA) was done separately for QoL variables, COVID‐specific scales, and appraisal items. If more than one composite was needed to maximize explained variance, a varimax rotation was implemented to ensure that composite scores were orthogonal and thus would not cause multicollinearity in subsequent multivariate modeling. Multivariate analysis of covariance (MANCOVA) was used to investigate group differences in QoL, COVID‐specific variables, and cognitive appraisal processes (dependent variables) as a function of group, comorbidity status, and their interaction, after adjusting for demographic characteristics. For these MANCOVA analyses, comorbidity status was operationalized as having four levels: zero, one, two, and three or more comorbidities. A Type I error rate of 0.05 was utilized for interpreting univariate results only if the multivariate effect was significant.^37^ Linear regression models were then implemented using hierarchical forward selection to investigate group differences in COVID‐specific variables (dependent variables) as a function of cognitive appraisal processes (entered first), PROs (entered first in next tranche), and demographic covariates (entered first in final tranche, followed by PROs, and finally appraisal composites).

IBM SPSS version 27^38^ was used for all analyses.

## 3 RESULTS

### 3.1 Sample

**FIGURE 1:**
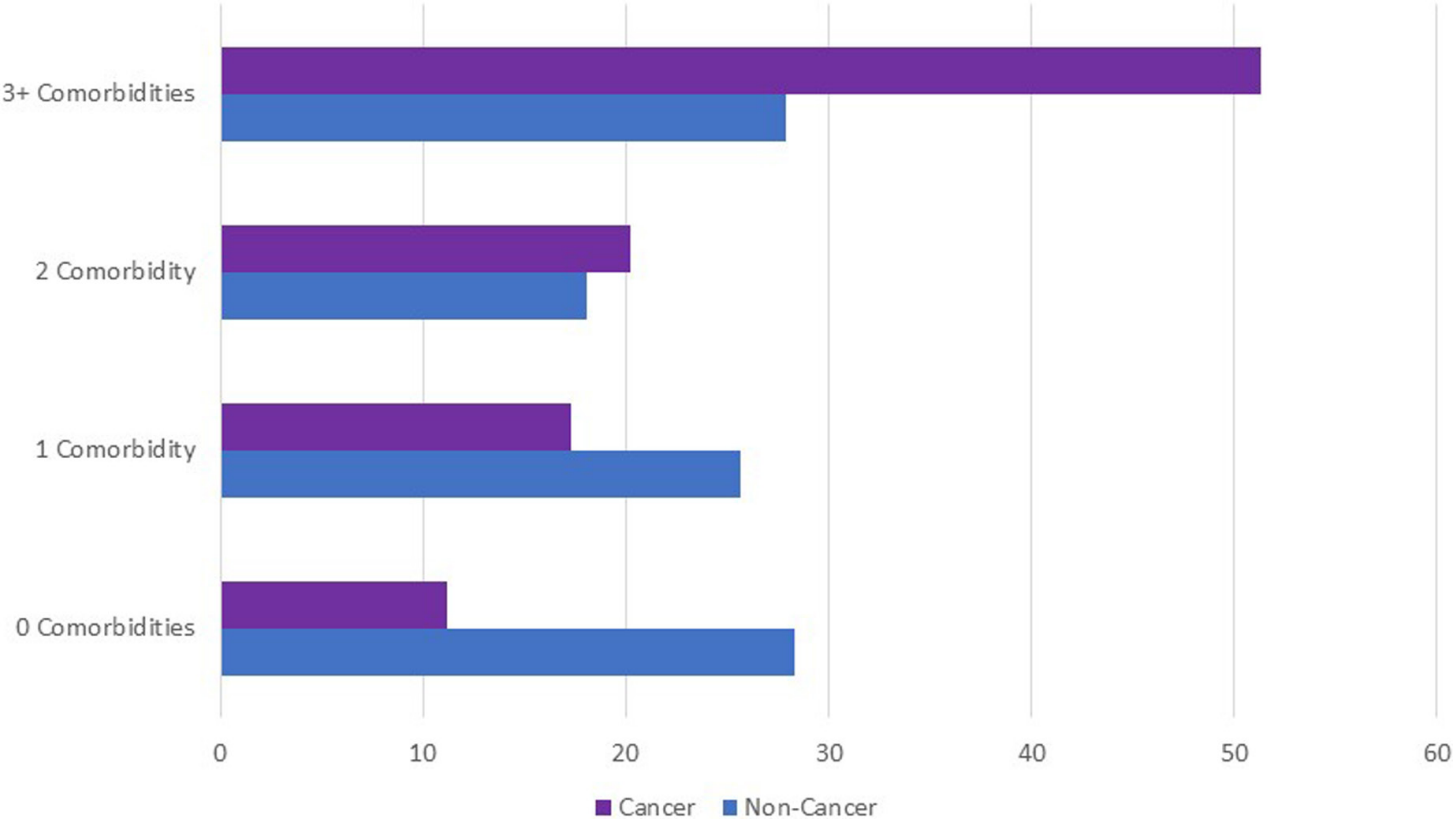
Number of comorbidities for cancer versus non‐cancer groups. Cancer survivors reported more comorbidities than non‐cancer participants across all groupings overall (*χ* ^2^ = 77.7, df = 3, *p* < 0.0001), and particularly in the 2, and 3+ groups (11% and 84% more).

Table 1 provides sociodemographic characteristics on the sample by cancer and non‐cancer groups (*n* = 520 and 441, respectively). The two groups were significantly different on almost every variable. Cancer survivors tended to be older, had more comorbid conditions, had been diagnosed more recently, and had a higher body mass index. They were more likely to be female, white, more educated, and less financially strained. They were more likely to live with a spouse or partner and not alone, less likely to smoke, and less likely to have had COVID‐19. In addition to reporting more comorbidities other than cancer (2.9 vs. 1.8), they were more likely to report having arthritis, back pain, depression, heart disease, insomnia, lung disease, or ulcer or stomach disease (Table 1). To avoid outlier effects and to enable examination of interaction effects, we categorized people according to numbers of comorbidities mentioned into four levels (0: 19%; 1: 21%, 2: 19%, 3 or more: 41%). Figure 1 displays cancer versus non‐cancer group differences in numbers of comorbidities.

**TABLE 1:** Demographic characteristics of cancer (*n* = 520) versus non‐cancer (*n* = 441) patients.

	Cancer patients		Non‐cancer patients		ANOVA comparison by group		
Variable	Mean^a^	Std. deviation	Mean	Std. deviation	*F* test	Error df	*p*
Age	60.88	10.37	52.28	17.89	86.21	959	<0.001
No. of comorbid conditions	2.87	2.11	1.80	1.88	67.66	959	<0.001
Years since diagnosis	9.90	7.33	16.22	14.57	59.27	719	<0.001
Body mass index	29.67	7.67	27.92	7.47	12.07	901	<0.001
					**Chi‐square**	**df**	***p***
What is your gender?	1.51	0.50	1.88	0.33	158.65	2	<0.001
White race	0.94	0.24	0.82	0.38	30.04	1	<0.001
Education level	2.80	1.04	2.49	1.06	28.77	3	<0.001
Difficulty paying bills	1.68	1.04	2.03	1.30	29.44	4	<0.001
Live with spouse/partner	0.71	0.45	0.53	0.50	34.31	1	<0.001
Alone	0.13	0.34	0.21	0.41	11.57	1	<0.001
Smoking status	0.11	0.31	0.30	0.46	55.37	1	<0.001
COVID‐19 infection status	0.05	0.23	0.13	0.34	17.67	1	<0.001
Comorbidities							
Arthritis	0.50	0.50	0.25	0.43	61.78	1	<0.001
Asthma	0.16	0.37	0.15	0.36	0.18	1	0.67
Back pain	0.51	0.50	0.31	0.46	37.25	1	<0.001
Cancer now or in the past	0.95	0.22	0.00	0.00	849.48	1	<0.001
Depression	0.42	0.49	0.24	0.43	33.87	1	<0.001
Diabetes	0.15	0.35	0.17	0.37	0.82	1	0.37
Heart disease	0.10	0.30	0.06	0.24	4.92	1	0.03
High blood pressure	0.35	0.48	0.33	0.47	0.53	1	0.47
Insomnia	0.33	0.47	0.12	0.33	57.05	1	<0.001
Kidney disease	0.07	0.25	0.04	0.20	3.14	1	0.08
Liver disease	0.03	0.18	0.02	0.15	0.85	1	0.36
Lung disease	0.12	0.32	0.05	0.21	14.51	1	<0.001
Stroke	0.03	0.18	0.04	0.20	0.29	1	0.59
Ulcer or stomach disease	0.12	0.33	0.04	0.19	21.61	1	<0.001

^a^ Means of binary variables reflect the proportion of the group endorsing this variable. These proportions were compared between groups using chi‐square.

### 3.2 Data reduction

The PCA of the QoL outcomes yielded one composite score summarizing the PROMIS physical and mental scores, the Ryff Environmental Mastery and Purpose in Life subscales, and the DQ Wellness score; and explained 65% of the variance (see Table S2; hereafter referred to as “QoL”). Neuro‐QoL Applied Cognition did not load onto the first component and was treated separately in multivariate analyses (“Cognitive Functioning Problems”).

The PCA of the COVID‐specific challenges/adaptations yielded four composite scores and explained 69% of the variance (see Table S3). The first composite comprised risk‐taking, hardship, and interpersonal conflict (“Risk/Hardship/Conflict”). The second comprised Post‐traumatic Growth and Social Support (“Growth/Support”). The third comprised self‐protection and emotional distress (“Protect/Distress”); and the fourth comprised altruism and coping with lockdown (“Altruism/Coping”).

The PCA of the appraisal items yielded six components that explained 60% of the variance (see Table S4). The first component comprised thinking a great deal about comparisons to other people or circumstances in order to evaluate their QoL (“Focused on Comparisons”); the second focused on problem goals (e.g., get out of a rut, feel settled, solve problem, get more help with multiple domains) (“Problem Goals”); the third on health goals (e.g., recent health problems and flare ups, get more support from providers, depend less on others) (“Health Goals”); the fourth on positive patterns of emphasis (e.g., emphasize the positive, get used to things) (“Positive Emphasis”); the fifth on negative patterns of emphasis (e.g., negative, out of control, resigned) (“Negative Emphasis”); and the sixth on sampling recent events, changes, and demands (“Recent Demands”).

### 3.3 Group differences in QoL

**FIGURE 2:**
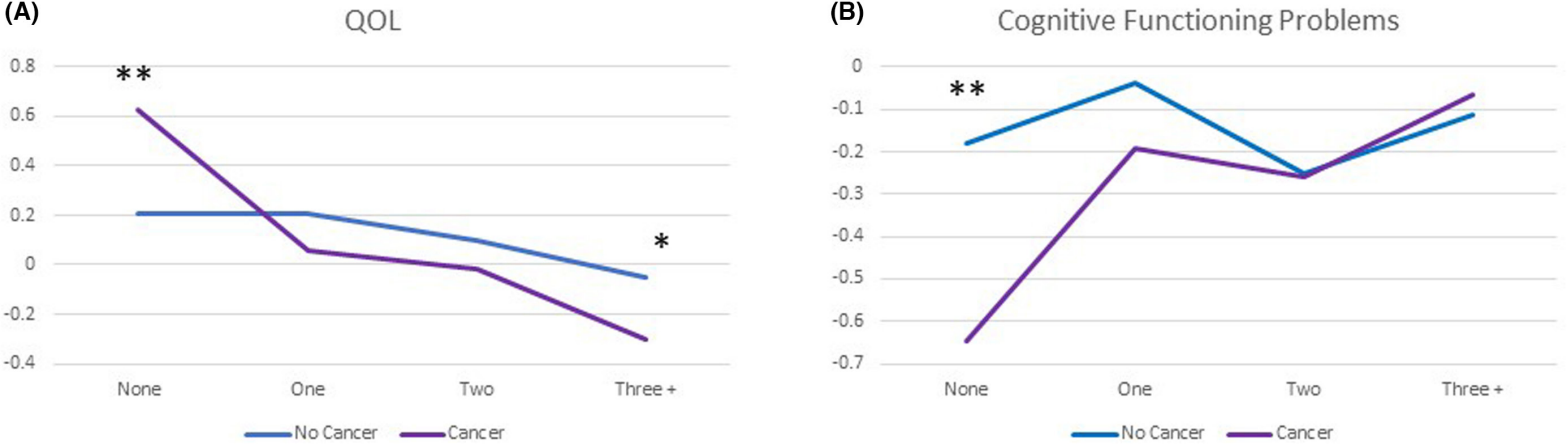
(A, B) Comparing the QoL composite and Neuro‐QoL Applied Cognition scores for cancer versus non‐cancer groups as a function of comorbidity level. As number of comorbidities increased, there was a downward trend in QoL and worsened cognitive functioning (*F*_Wilk's Lambda_ = 8.6, df = 6, *p* < 0.0001). Significant interactions between comorbidity level and cancer group for both the QoL and Applied Cognition outcomes (*F*_multivariate_ = 5.0 and 3.2, *p* = 0.002 and 0.023, respectively) revealed group differences on QoL among those with zero comorbidities and three or more comorbidities (post hoc comparison *p* = 0.006 and 0.019, respectively); and on Applied Cognition among those with zero comorbidities (post hoc comparison *p* = 0.002). Asterisks highlight significant interaction effects (**p < 0.01, *p < 0.05).

Results of MANCOVA models revealed significant interactions between comorbidity level and cancer group for both QoL and cognitive functioning problems (Figure 2A,B; Tables S5 and S6). Cancer survivors fared substantially better than non‐cancer participants in QoL and cognitive functioning when they had no other comorbidities, and substantially worse on QoL when they had three or more comorbidities (Figure 2A), and on cognitive functioning problems among those with zero comorbidities (Figure 2B). Significant covariates included education level, difficulty paying bills, and living with a spouse/partner.

### 3.4 Group differences in COVID‐specific variables

**FIGURE 3:**
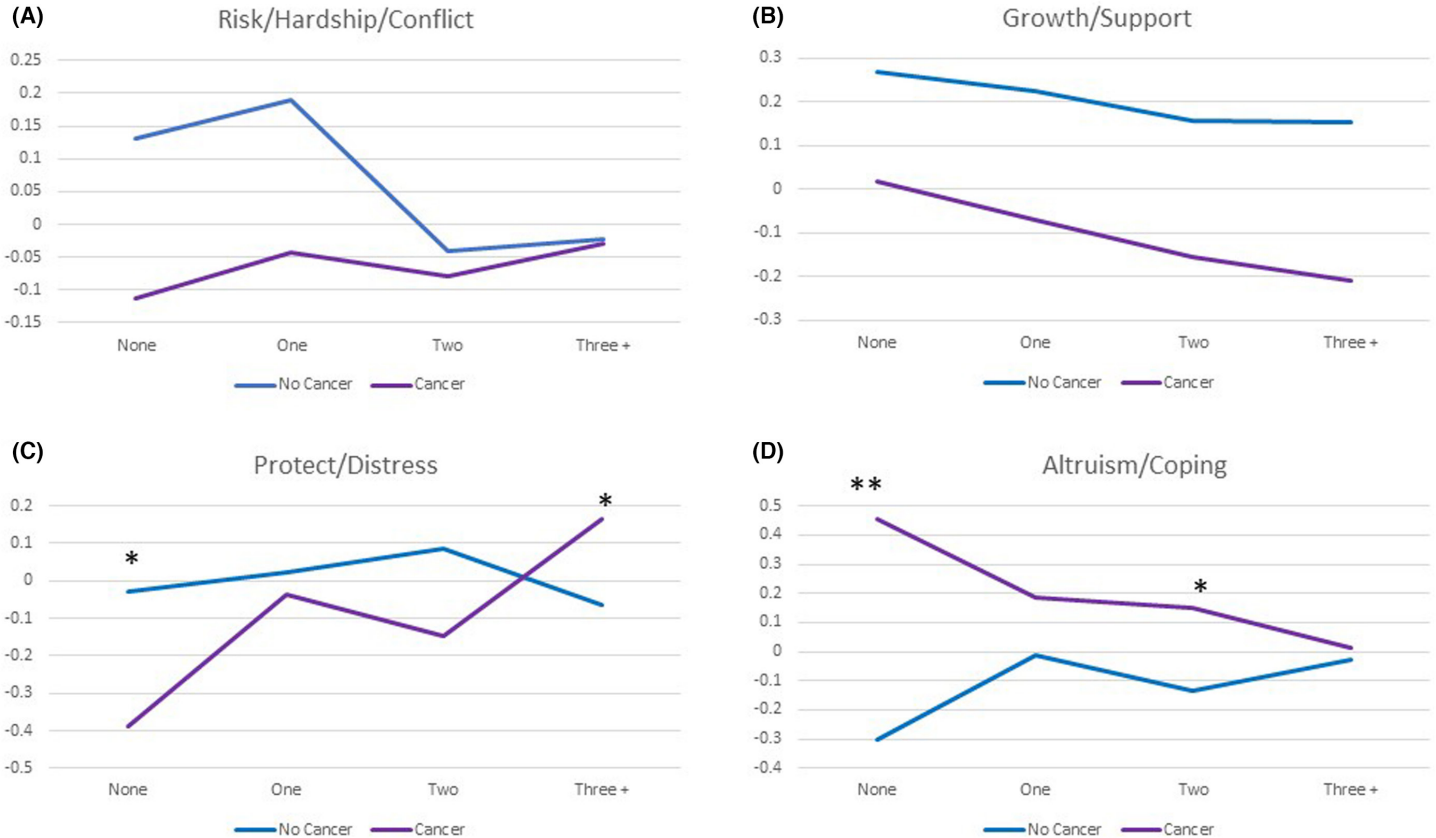
(A–D) Comparing COVID‐specific composite scores for cancer versus non‐cancer groups as a function of comorbidity level. Cancer survivors differed from non‐cancer participants on the COVID‐specific composite scores both overall (*F*_Wilk's Lambda_ = 14.6, df = 4, *p* < 0.0001) and as a function of comorbidity level (*F*_Wilk's Lambda_ = 2.6, df = 12, *p* = 0.002). Cancer survivors reported less growth/social support overall (*F*_Between‐subjects_ = 14.8, *p* < 0.0001), compared to non‐cancer participants. There was a significant interaction between cancer group and comorbidity level for the COVID protection and worry and covid altruism and coping composite scores (*F*_Between‐subjects_ = 4.2 and 5.4, *p* = 0.006 and 0.001, respectively). Post hoc comparisons revealed significant group differences on COVID protection and worry among those with zero comorbidities and three or more comorbidities (*p* = 0.025 and 0.039, respectively), and on COVID altruism and coping among those with zero and two comorbidities (*p* < 0.0001 and 0.05, respectively). Asterisks highlight significant interaction effects (**p < 0.01, *p < 0.05).

Results of MANCOVA models predicting the COVID composite scores revealed a significant interaction between comorbidity level and cancer group overall, after adjusting for covariates (Figure 3A–D; Tables S5 and S7). Cancer survivors with no comorbidities fared substantially better than non‐cancer participants on protection and worry and on altruism and coping, but substantially worse when they had three or more or two comorbidities, respectively. There were significant main effects of cancer versus non‐cancer on growth/support (*p* < 0.0001) suggesting that independent of the interaction with comorbidities, cancer survivors were less likely overall to report enhanced growth/support and more likely to engage in altruistic/positive coping at this early time in the pandemic. Significant covariates included gender, white race, education level, difficulty paying bills, living with a spouse/partner, and living alone.

### 3.5 Group differences in cognitive‐appraisal processes

**FIGURE 4:**
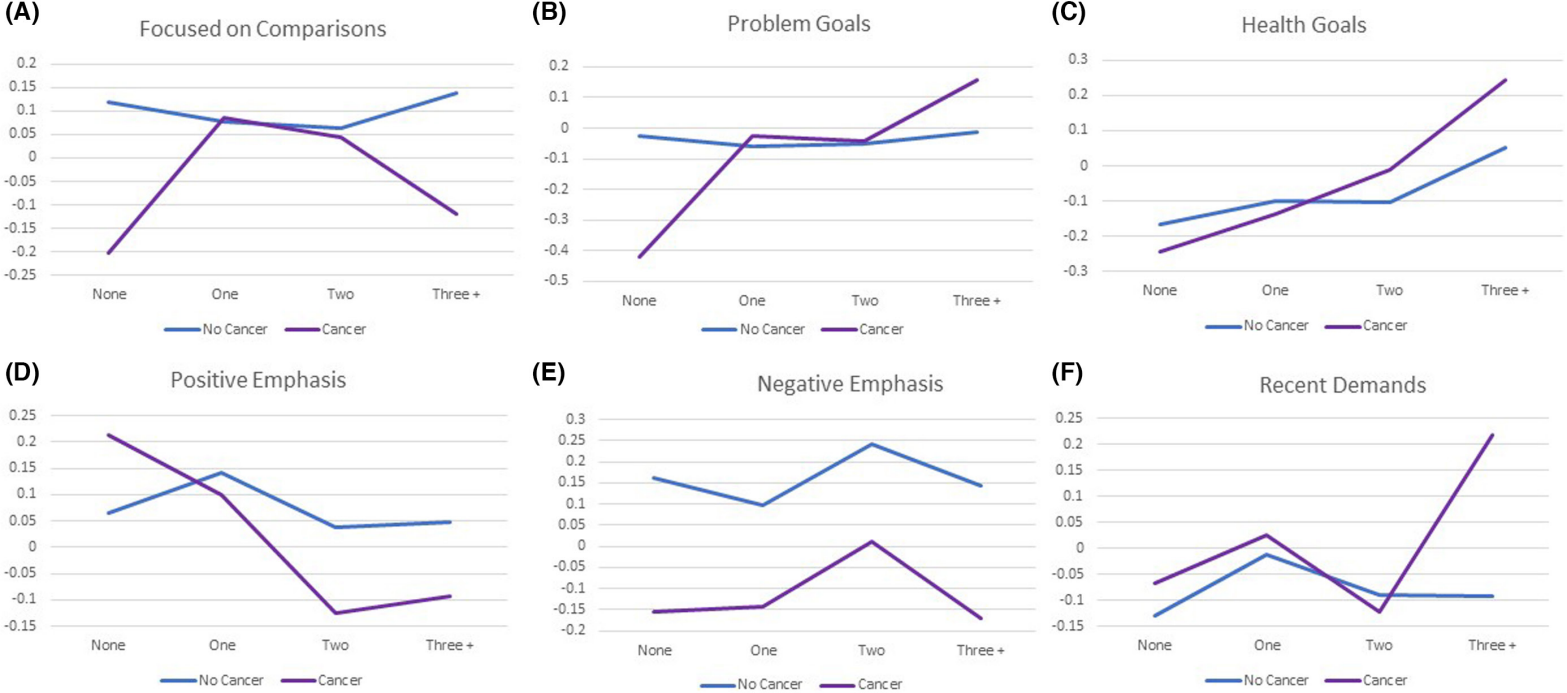
(A–F) Comparing appraisal composite scores for cancer versus non‐cancer groups as a function of comorbidity level. Cancer survivors differed from non‐cancer participants on the appraisal composite scores (*F*_Wilk's Lambda_ = 3.3, df = 6, *p* = 0.003) as did comorbidity levels (*F*_Wilk's Lambda_ = 2.5, df = 18, *p* < 0.0001). Cancer survivors generally engaged in fewer negative‐emphasis appraisals than non‐cancer participants (*F*_Between‐subjects_ = 12.6, *p* < 0.0001, E). As number of comorbidities increased, problem goals and health goals also increased overall (*F*_Between‐subjects_ = 3.5 and 5.4, *p* = 0.014 and 0.001, respectively, B, C).

Results of MANCOVA models predicting the appraisal composite scores revealed significant main effects for cancer group and comorbidity level, but no significant interaction, after adjusting for covariates (Figure 4A–F; Tables S5 and S8). The non‐cancer group was more focused on the negative (Figure 4E). There was also a significant main effect for comorbidity level on problem goals and health goals, such that as one experienced increasing comorbidities, goals related to health and to other problems became more salient (Figure 4B,C). Significant covariates included gender, education level, difficulty paying bills, and living with a spouse/partner.

### 3.6 Group differences in COVID‐specific variables as a function of cognitive‐appraisal processes

The above results revealed that cancer survivors responded to COVID in different ways than non‐cancer participants, and that the role of comorbidities appeared to differ. Accordingly, the next series of analyses examined whether these cancer versus non‐cancer differences in the impact of COVID were attributable to ways of appraising QoL over and above level of QoL, after adjusting for relevant demographic characteristics.

#### 3.6.1 Predictors of risk/hardship/conflict

For cancer survivors, COVID‐specific hardship was related to worse financial difficulty, lower education, and worse QoL (Table S9). Appraisal processes characterized by focusing on comparisons, negative emphasis, problem goals, and recent demands were all related to worse hardship. Significant interaction effects all related to QoL such that those with a low QoL had worse hardship, and this impact was even greater for those who emphasized the negative, focused on problem goals, and on recent demands.

For the comparison group, worse hardship was reported by those with worse financial difficulty. In contrast to cancer survivors, the more educated people in the comparison group reported worse hardship. Younger people and those with worse QoL reported worse hardship. Similar to cancer survivors, comparison participants who focused on comparisons, negative emphasis, and recent demands reported worse hardship. Additionally, those with more comorbidities who emphasized the positive had worse hardship.

#### 3.6.2 Predictors of growth/support

For cancer survivors, reporting more COVID‐specific post‐traumatic growth and social support was related to living with a spouse/partner, having more comorbidities, and better QoL (Table S9). Appraisal processes characterized by focusing on comparisons, health goals, and emphasizing the positive were all associated with greater growth/support. Those with better QoL who focused on comparisons reported lower growth/support, whereas those with more comorbidities who also endorsed more problem goals had higher growth/support.

For the comparison group, reporting more COVID‐specific growth/support was related to living with a spouse/partner, and reporting better QoL (Table S9). Appraisal processes characterized by focusing on comparisons, health goals, and emphasizing the positive were all associated with greater growth/support.

#### 3.6.3 Predictors of protect/distress

For cancer survivors, COVID‐specific self‐protection and worry was related to worse financial difficulty, and worse QoL (Table S9). People who were doing more actions to protect themselves from COVID and experienced more anxiety specifically about the pandemic tended to focus on recent demands rather than dwelling on the negative. Those with fewer comorbidities who tended to emphasize the positive were less worried and less engaged in protective actions.

For the comparison group, COVID‐specific protect/distress was similarly related to worse financial difficulty, and worse QoL (Table S9). Appraisal processes characterized by focusing on comparisons, health goals, and recent demands, and not emphasizing the negative, were related to worse protect/distress. Those with worse QoL who also focused on recent demands reported lower protect/distress.

#### 3.6.4 Predictors of altruism/coping

For cancer survivors, reporting more COVID‐specific altruism and positive coping approaches were related to less financial difficulties, higher education, living with a spouse/partner, and reporting a better QoL (Table S9). Appraisal processes characterized by not emphasizing the negative were associated with more altruism/coping.

For the comparison group, reporting more COVID‐specific altruism and positive coping approaches were related to higher education, reporting more comorbidities, but also a better QoL (Table S9). Appraisal processes characterized by focusing on comparisons, and emphasizing the negative were associated with more altruism/coping. Those with more comorbidities who also focused more on recent demands also reported higher levels of altruism/coping.

**FIGURE 5:**
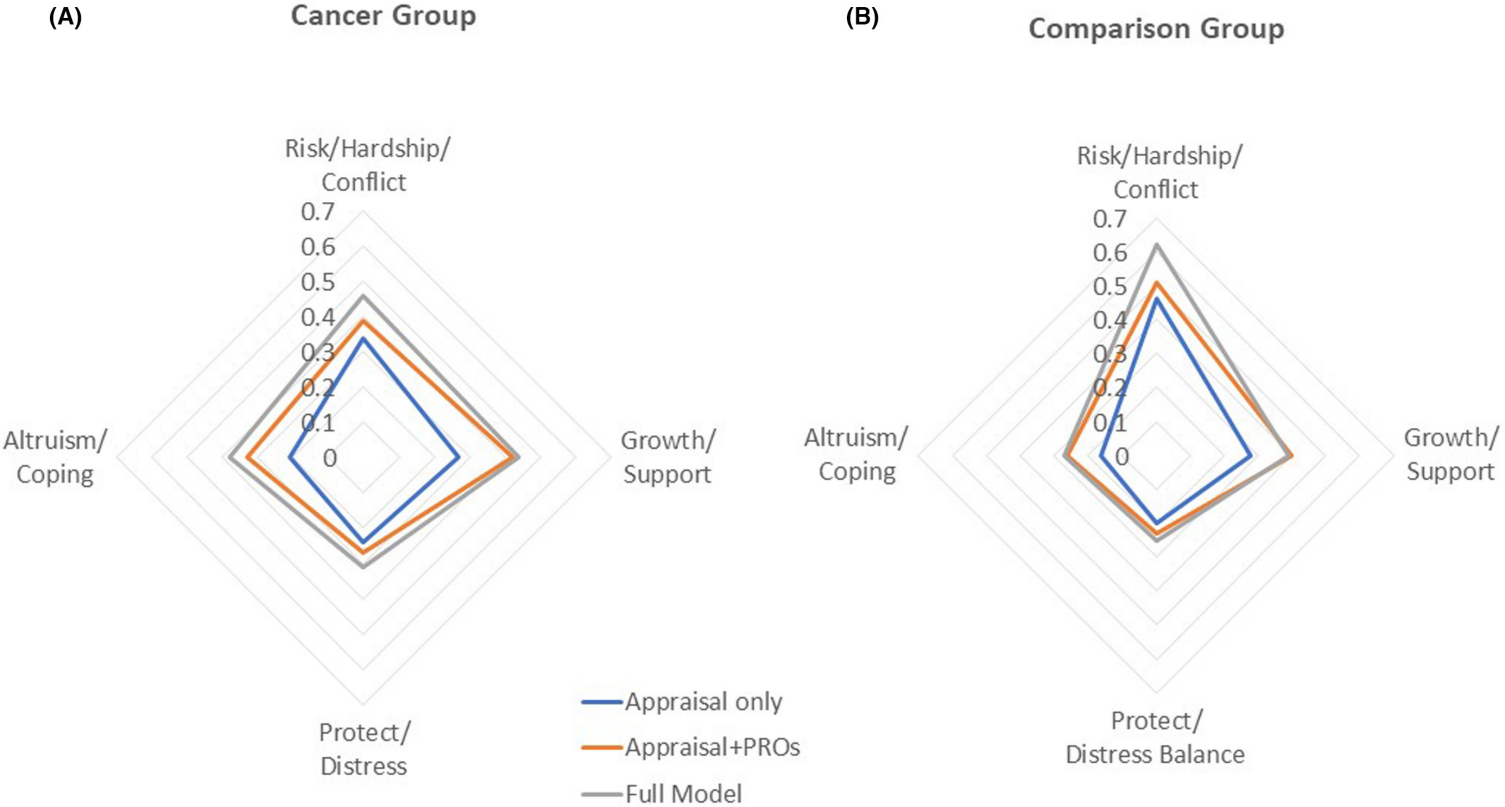
(A, B) Radar charts showing explained variance in the three sets of models for cancer versus non‐cancer comparison groups. This components‐of‐variance finding suggests that the impact of demographic variables, comorbidity status, and QoL on COVID‐specific variables can be explained because one appraises differently.

Appraisal alone explained a large proportion of the variance in COVID‐specific variables compared to comorbidity, QoL, and demographic characteristics. Figure 5A,B illustrate this point using radar plots that show explained variance in the appraisal only models, appraisals plus PROs (comorbidity, QoL), and the full models (appraisal plus PROs and demographics). For the cancer and comparison groups, appraisal explained about three‐quarters of the explained variance in risk/hardship/conflict, growth/support, and protect/distress, and over half of the variance in altruism/coping. This components‐of‐variance finding suggests that the impact of demographic variables, comorbidity status, and QoL on COVID‐specific variables can be explained because one appraises differently.

## 4 DISCUSSION

Cancer survivors' experiences of the early pandemic were markedly different than people who were not affected by cancer, particularly when comorbidities and other life circumstances were considered. Specifically, cancer survivors fared substantially better than others in QoL and cognitive functioning when they had no other comorbidities, but substantially worse on QoL when they had three or more comorbidities. Survivors were also less likely overall to report a sense of post‐traumatic growth or receiving social support (i.e., growth/support), but more likely to provide tangible support to friends, family, and community, and salutogenic self‐care (i.e., altruism/coping). Cancer survivors also reported lower levels of protect/distress compared to non‐cancer participants, unless they had multiple comorbidities in addition to cancer. Finally, cancer survivors were less likely to engage in negative‐emphasis appraisals. Survivors thus seemed to have a greater equanimity in the face of the global pandemic. These findings might suggest an adaptive style of cancer survivors such that they prioritize engaging in problem‐focused, prosocial actions unless confronted with multiple comorbidities, at which point they exhibit more proactive self‐protection and experience more anxiety about the pandemic. These findings are reminiscent of the Folkman and Lazarus distinction of emotion‐focused versus problem‐focused coping.^39^

Groups also showed some differences in how demographic and appraisal processes were associated with COVID‐specific challenges/adaptations. Table S10 provides a summary schematic of these results to highlight similarities and differences across groups. While similar appraisal processes were positively associated with risk/hardship/conflict, growth/support, and protect/distress, there were notable differences in altruism/coping, and more appraisal processes came into play for these latter two outcomes for non‐cancer participants. Most notable were that altruism/coping was associated with not emphasizing the negative among survivors, but the opposite for non‐cancer participants. There were also notable group differences in the role of age, education, and difficulty paying bills.

We were surprised that an emphasis on the negative was associated with altruism and coping behavior in different directions for the cancer versus the non‐cancer groups (less and more likely, respectively). These differences may reflect how both older age and having (had) cancer can impart greater equanimity. Being older may also allow more flexibility for doing altruistic activities because child‐care responsibilities are less or no longer present.

The present work has the advantage of a large enough sample size to support substantial multivariate analyses that include interactions. It also teaches us more about appraisal, specifically that there are qualitative differences in meaning depending on one's context (e.g., age, health status, etc.). The limitations of the present work include a sample with more Whites than non‐Whites, which may limit the generalizability of the study findings. Additionally, we are not able to calculate a response rate given the participant‐recruitment sources, again possibly limiting the generalizability of the findings. A counter to this possible limit to generalizability is that the Ipsos comparison sample was specifically recruited to be representative of the adult population in the United States. A further possible limitation is that the COVID‐specific measures were derived from recommended individual items from the NIH rather than scales developed from rigorous psychometric testing. Of note, these scales had lower alpha reliability than scales that are commonly used in PRO research. Lower internal consistency reliability may have attenuated observed associations between study variables. Another limitation relates to the fact that the models were built from cross‐sectional data, thereby restricting causal inference. Such a limitation may be addressed using longitudinal data, although the quasi‐experimental nature of the study data would prevent explicit causal inference. Although adjusting for demographic factors has helped us isolate group differences specific to the comorbidity‐impact research question, it is possible that people's reaction to the pandemic may not only be dependent on having cancer versus not having cancer with or without comorbidities. Even multivariate control does not fully neutralize sizable preexisting group differences.^40^ Future work might utilize a matched sample to compare cancer and non‐cancer participants. A final limitation is that the timing of the data collection was relatively early in the pandemic. Although associations observed here may have evolved over time, this study affords a look at the impact of COVID when the crisis was highly threatening and very poorly understood.

### 4.1 Conclusions

In summary, cancer survivors had important differences from the comparison group in the impact of comorbidities on QoL and COVID‐specific challenges/adaptations. Appraisal processes also differed across groups as a function of number of comorbidities. Thus, the impact of having multiple comorbidities in the context of cancer is associated with notable differences in social determinants of health, QoL outcomes, COVID‐specific challenges/adaptations, and the one's appraisal of QoL. These findings provide an empirical basis for implementing appraisal‐based interventions (e.g., cognitive‐behavioral therapy^41^ or more targeted public health messaging^42^, ^43^) to help people cope with health challenges in general, and particularly in the context of the COVID pandemic.

## AUTHOR CONTRIBUTIONS

**Carolyn E. Schwartz:** Conceptualization (equal); data curation (equal); formal analysis (equal); validation (equal); visualization (supporting); writing – original draft (supporting); writing – review and editing (equal). **Bruce D. Rapkin:** Conceptualization (equal); data curation (equal); formal analysis (equal); validation (equal); visualization (supporting); writing – original draft (supporting); writing – review and editing (equal).

## FUNDING INFORMATION

This work was not funded by an external organization.

## CONFLICT OF INTEREST STATEMENT

Both authors declare that they have no potential conflicts of interest and report no disclosures.

## CODE AVAILABILITY

Requests for software code will be considered and will be made available if deemed reasonable.

## ETHICS STATEMENT

This study was conducted in accordance with the provision of the Declaration of Helsinki. The study was approved by the New England Independent Review Board (NEIRB #2021164).

## CONSENT TO PARTICIPATE

All the patients provided written informed consent before participation.

## CONSENT TO PUBLISH

All participants agreed to their data being published in a journal article.

## Supporting information


Data S1.

## Data Availability

The study data are confidential and thus not able to be shared.

## Endnote

* This question was worded as follows: “In what year (YYYY) were you diagnosed with your primary health condition?” For cancer patients, it would likely refer to date of cancer diagnosis, whereas for the general population sample, it would refer to the year of diagnosis with their most prominent comorbidity.

## References

[1] Zhou F , Yu T , Du R , et al. Clinical course and risk factors for mortality of adult inpatients with COVID‐19 in Wuhan, China: a retrospective cohort study. Lancet. 2020;395(10229):1054‐1062. 32171076 10.1016/S0140-6736(20)30566-3 PMC7270627

[2] Willems SJ , Castells MC , Baptist AP . The magnification of health disparities during the COVID‐19 pandemic. J Allergy Clin Immunol Pract. 2022;10(4):903‐908. 35131511 10.1016/j.jaip.2022.01.032 PMC8813673

[3] Gravlee CC . Systemic racism, chronic health inequities, and COVID‐19: a syndemic in the making? Am J Hum Biol. 2020;32:e23482. 32754945 10.1002/ajhb.23482 PMC7441277

[4] Kim EJ , Marrast L , Conigliaro J . COVID‐19: magnifying the effect of health disparities. J Gen Intern Med. 2020;35(8):2441‐2442. 32394141 10.1007/s11606-020-05881-4 PMC7213773

[5] Barrett ADT , Titball RW , MacAry PA , et al. The rapid progress in COVID vaccine development and implementation. NPJ Vaccines. 2022;7(1):20. 35145102 10.1038/s41541-022-00442-8 PMC8831501

[6] Singh M , de Wit E . Antiviral agents for the treatment of COVID‐19: progress and challenges. Cell Rep Med. 2022;3(3):100549. 35474740 10.1016/j.xcrm.2022.100549 PMC8831133

[7] Malik P , Patel K , Pinto C , et al. Post‐acute COVID‐19 syndrome (PCS) and health‐related quality of life (HRQoL)—a systematic review and meta‐analysis. J Med Virol. 2022;94(1):253‐262. 34463956 10.1002/jmv.27309 PMC8662132

[8] Kompaniyets L , Pennington AF , Goodman AB , et al. Peer reviewed: underlying medical conditions and severe illness among 540,667 adults hospitalized with COVID‐19, March 2020–March 2021. Prev Chronic Dis. 2021;18:E66. 34197283 10.5888/pcd18.210123 PMC8269743

[9] Addeo A , Friedlaender A . Cancer and COVID‐19: unmasking their ties. Cancer Treat Rev. 2020;88:102041. 32516704 10.1016/j.ctrv.2020.102041 PMC7831797

[10] Tang K , Wei Z , Wu X . Impaired serological response to COVID‐19 vaccination following anticancer therapy: a systematic review and meta‐analysis. J Med Virol. 2022;94(10):4860‐4868. 35750492 10.1002/jmv.27956 PMC9349696

[11] Johannsen A , Susin C , Gustafsson A . Smoking and inflammation: evidence for a synergistic role in chronic disease. Periodontol 2000. 2014;64(1):111‐126. 24320959 10.1111/j.1600-0757.2012.00456.x

[12] Centers for Disease Control and Prevention (CDC) . Cigarette smoking among adults—United States, 2006. MMWR Morb Mortal Wkly Rep. 2007;56(44):1157‐1161. 17989644

[13] Lawton MP , Nahemow L . Ecology and the aging process. In: Eisdorfer C , Lawton MP , eds. The Psychology of Adult Development and Aging. American Psychological Association; 1973:619‐674.

[14] Dubey S , Biswas P , Ghosh R , et al. Psychosocial impact of COVID‐19. Diabetes Metab Syndr Clin Res Rev. 2020;14(5):779‐788. 10.1016/j.dsx.2020.05.035 PMC7255207 32526627

[15] Amaniera I , Bach C , Vachani C , et al. Psychosocial impact of the COVID‐19 pandemic on cancer patients, survivors and caregivers. J Psychosoc Oncol. 2021;39(3):485‐492. 33870877 10.1080/07347332.2021.1913780

[16] Park CL , Russell BS , Fendrich M , Finkelstein‐Fox L , Hutchison M , Becker J . Americans' COVID‐19 stress, coping, and adherence to CDC guidelines. J Gen Intern Med. 2020;35(8):2296‐2303. 32472486 10.1007/s11606-020-05898-9 PMC7259430

[17] Park CL , Finkelstein‐Fox L , Russell BS , Fendrich M , Hutchison M , Becker J . Americans' distress early in the COVID‐19 pandemic: protective resources and coping strategies. Psychol Trauma Theory Res Pract Policy. 2021;13(4):422‐431. 10.1037/tra0000931 PMC8448577 33507795

[18] Schwartz CE , Snook E , Quaranto B , Benedict RH , Rapkin BD , Vollmer T . Cognitive reserve and appraisal in multiple sclerosis. Mult Scler Relat Disord. 2013;2(1):36‐44. 25877453 10.1016/j.msard.2012.07.006

[19] Schwartz CE , Finkelstein JA , Rapkin BD . Appraisal assessment in patient‐reported outcome research: methods for uncovering the personal context and meaning of quality of life. Qual Life Res. 2017;26(26):545‐554. 27988907 10.1007/s11136-016-1476-2

[20] Schwartz CE , Stark RB , Rapkin BD . Capturing patient experience: does quality‐of‐life appraisal entail a new class of measurement? J Patient Rep Outcomes. 2020;4:85. 33108540 10.1186/s41687-020-00254-1 PMC7591682

[21] Rapkin BD , Schwartz CE . Advancing quality‐of‐life research by deepening our understanding of response shift: a unifying theory of appraisal. Qual Life Res. 2019;28(10):2623‐2630. 31321672 10.1007/s11136-019-02248-z

[22] Rapkin BD , Schwartz CE . Toward a theoretical model of quality‐of‐life appraisal: implications of findings from studies of response shift. Health Qual Life Outcomes. 2004;2(1):14. 15023229 10.1186/1477-7525-2-14 PMC408464

[23] Hays RD , Bjorner JB , Revicki DA , Spritzer KL , Cella D . Development of physical and mental health summary scores from the patient‐reported outcomes measurement information system (PROMIS) global items. Qual Life Res. 2009;18:873‐880. 19543809 10.1007/s11136-009-9496-9 PMC2724630

[24] Cella D , Lai JS , Nowinski C , et al. Neuro‐QOL: brief measures of health‐related quality of life for clinical research in neurology. Neurology. 2012;78:1860‐1867. 22573626 10.1212/WNL.0b013e318258f744 PMC3369516

[25] Ryff CD . Happiness is everything, or is it? Explorations on the meaning of psychological well‐being. J Pers Soc Psychol. 1989;57:1069‐1081.

[26] Ryff CD . Psychological well‐being revisited: advances in the science and practice of eudaimonia. Psychother Psychosom. 2014;83(1):10‐28. 24281296 10.1159/000353263 PMC4241300

[27] Schwartz CE , Stucky BD , Stark RB . Operationalizing the attitudes, behaviors, and perspectives of wellness: a brief measure for use in resilience research. Health Psychol Behav Med. 2021;9(1):1031‐1052. 34881116 10.1080/21642850.2021.2008940 PMC8648008

[28] NIH Office of Behavioral and Social Sciences Research (OBSSR), NIH Disaster Research Program (DR2) . COVID‐19 BSSR Research Tools. 1st ed. NIH Office of Behavioral and Social Sciences Research (OBSSR); 2020.

[29] Tedeschi RG , Calhoun LG . The posttraumatic growth inventory: measuring the positive legacy of trauma. J Trauma Stress. 1996;9:455‐471. 8827649 10.1007/BF02103658

[30] Tedeschi RG , Cann A , Taku K , Senol‐Durak E , Calhoun LG . The posttraumatic growth inventory: a revision integrating existential and spiritual change. J Trauma Stress. 2017;30(1):11‐18. 28099764 10.1002/jts.22155

[31] Tedeschi RG , Moore BA . Posttraumatic growth as an integrative therapeutic philosophy. J Psychother Integr. 2021;31(2):180‐194.

[32] Rapkin BD , Garcia I , Michael W , Zhang J , Schwartz CE . Distinguishing appraisal and personality influences on quality of life in chronic illness: introducing the quality‐of‐life appraisal profile version 2. Qual Life Res. 2017;26:2815‐2829. 28593531 10.1007/s11136-017-1600-y

[33] Schwartz CE , Stucky BD , Michael W , Rapkin BD . Does response shift impact interpretation of change even among scales developed using item response theory? J Patient Rep Outcomes. 2020;4(1):8. 31975159 10.1186/s41687-019-0162-x PMC6977794

[34] Schwartz CE , Stark RB , Rapkin BD , Selbe S , Michael W , Stopka T . Cognitive habits linked to resilience: surprising commonalities across the United States. J Community Med Public Health Rep. 2020;1(1):1‐14.

[35] Li Y , Rapkin BD . Classification and regression tree analysis to identify complex cognitive paths underlying quality of life response shifts: a study of individuals living with HIV/AIDS. J Clin Epidemiol. 2009;62:1138‐1147. 19595576 10.1016/j.jclinepi.2009.03.021 PMC2754600

[36] Schwartz CE , Zhang J , Rapkin BD , Finkelstein JA . Reconsidering the minimally important difference: evidence of instability over time and across groups. Spine J. 2019;19(4):726‐734. 30248391 10.1016/j.spinee.2018.09.010

[37] Tabachnick BG , Fidell LS , Ullman JB . Using Multivariate Statistics. Pearson; 2007.

[38] IBM , ed. IBM SPSS Statistics for Windows. 28th ed. IBM Corp; 2021.

[39] Folkman S , Lazarus RS , Gruen RJ , DeLongis A . Appraisal, coping, health status, and psychological symptoms. J Pers Soc Psychol. 1986;50(3):571‐579. 3701593 10.1037//0022-3514.50.3.571

[40] Miller GA , Chapman JP . Misunderstanding analysis of covariance. J Abnormal Psych. 2001;110(1):40‐48. 10.1037//0021-843x.110.1.40 11261398

[41] McGinn LK . Cognitive behavioral therapy of depression: theory, treatment, and empirical status. Am J Psychother. 2000;54(2):257‐262. 10928248 10.1176/appi.psychotherapy.2000.54.2.257

[42] Merchant RM , South EC , Lurie N . Public health messaging in an era of social media. JAMA. 2021;325(3):223‐224. 33393964 10.1001/jama.2020.24514

[43] Lang R , Benham JL , Atabati O , et al. Attitudes, behaviours and barriers to public health measures for COVID‐19: a survey to inform public health messaging. BMC Public Health. 2021;21(1):1‐15. 33882896 10.1186/s12889-021-10790-0 PMC8058588

