# Supplementary material for: Impact of comorbidities on people with and without cancer early in the COVID‐19 pandemic: An observational study
Source: Cancer Med. 2023 Jun 3;12(14):15601–11. doi: 10.1002/cam4.6212 (PMC10417288; doi:10.1002/cam4.6212)
Supplement: Supplementary file 1 — Data S1. [file CAM4-12-15601-s001.pdf]

Supplemental Table 1. COVID-Specific Items and Scales

Concept	Item	Time Frame	Alpha coefficient†
COVID Infection Status	Have you had COVID-19, the illness caused by the novel coronavirus?	Currently	NA
Self-Protection	Washing hands and/or using sanitizer after returning home	Currently	0.75
	Staying at least 6 feet away from others outside the home	Currently	
	Wearing a face mask in public	Currently	
	Wiping down surfaces with disinfectant	Currently	
Risk-Taking	Gone to a grocery store or pharmacy	Past 7 days	0.60
	Gotten together with a friend, neighbor, or relative	Past 7 days	
	Went to a restaurant or bar or attended a gathering with more than 10 people	Past 7 days	
	Sought healthcare for illness or injury other than COVID	Past 7 days	
	Shared items like towels, forks or spoons with other people	Past 7 days	
Hardship	Not enough money to pay rent or mortgage	Currently	0.88
	Not enough money to pay for gas	Currently	
	Not enough money to pay for food	Currently	
	Did not have a regular place to sleep or stay	Currently	
	Losing my job	Future concern	
	Another family member losing his/her job	Future concern	
	Not enough money to pay rent or mortgage	Future concern	
	Not enough money to pay for gas	Future concern	
	Not enough money to pay for food	Future concern	
	Not having a regular place to sleep or stay	Future concern	
	Difficulty getting the usual medicine that you need	During the pandemic	
	Difficulty getting routine medical care	During the pandemic	
	Have delays or difficulties reaching your healthcare provider(s) caused your health to worsen in any way?	During the pandemic	
Interpersonal Conflict	Arguments with your spouse or partner	Past 7 days	0.76
	Snapping at or yelling at other family members or loved ones	Past 7 days	
	Getting angry at children	Past 7 days	
	Interpersonal conflict with friends or coworkers	Past 7 days	
Emotional Distress	How often do you feel isolated from others?	Currently	0.62
	How often do you feel irritable or grumpy?	Currently	
	I am not worried about the coronavirus (rec)	Currently	
	I am stressed around other people because I worry I'll catch the coronavirus	Currently	
	Thoughts about the pandemic distract me or keep me from being able to concentrate	Currently	
Post-Traumatic Growth	I have a greater appreciation for the value of my own life	Since the pandemic began	0.82
	I more clearly see that I can count on people in times of trouble	Since the pandemic began	
	I have a greater sense of closeness with others	Since the pandemic began	
	I have a stronger religious faith	Since the pandemic began	
Altruism	Brought food or medicine to neighbors	Past month	0.35
	Donated money	Past month	
	Donated blood	Past month	
	Made contacts with friends or family to keep in touch	Past month	
	Helped someone with child care	Past month	
Coping with Lockdown	Taking breaks from watching, reading, or listening to news stories, including social media	Past 7 days	0.58
	Eating healthy, well-d meals	Past 7 days	
	Eating a lot of high-fat or sugary foods	Past 7 days	
	Exercising for at least 20 minutes	Past 7 days	
	Getting enough sleep	Past 7 days	
Social Support	To listen to you when you needed to talk?	Currently	0.58
	To have a good time with?	Currently	
	To turn to for suggestions about how to deal with a personal problem?	Currently	
	To understand your problems?	Currently	
	To love and make you feel wanted?	Currently	
	How many supports in the home	Currently	

** Reverse-coded*

† Computed on whole sample.

Supplemental Table 2. Results of Principal Components Analyses for QOL Variables

Variable	Component Loading
PROMIS-10 Mental (T-score)	**0.82**
PROMIS-10 Physical (T-score)	**0.74**
Ryff Environmental Mastery	**0.84**
Ryff Purpose in Life	**0.78**
DeltaQuest Wellness	**0.85**
Eigenvalue	3.27
Cumulative Variance Explained	65.36

Note: Bolded numbers have loadings of 0.40 or greater.

Supplemental Table 3. Results of Principal Components Analyses for COVID-Specific Variables

Variable	Varimax Rotation Component Loading 1 Risk/Hardship/ Conflict	2 Growth/ Support	3 Protect/ Distress	4 Altruism/ Coping
Self-Protection	-0.11	0.27	**0.74**	0.18
Risk-Taking (high=greater risk)	**0.76**	0.05	-0.09	0.13
Hardship at Follow-Up	**0.74**	-0.07	0.43	-0.27
Post-Traumatic Growth	0.18	**0.79**	0.28	0.09
Altruism	0.39	0.10	0.24	**0.62**
Social Support	-0.13	**0.78**	-0.22	0.44
Interpersonal Conflict	**0.81**	-0.01	0.20	-0.02
Emotional Distress	0.49	-0.28	**0.74**	-0.23
Coping with Lockdown	-0.24	0.32	-0.12	**0.79**
Eigenvalue	2.37	1.75	1.24	0.86
% of Variance Explained	26.37	19.44	13.81	9.57
Cumulative Variance Explained				69.19

Note: Bolded numbers have loadings of 0.40 or greater.

Supplemental Table 4. Results of Principal Components Analyses for Appraisal Variables						
	Varimax Component Loading					
	1	2	3	4	5	6
Variable	Focused on Comparisons	Problem Goals	Health Goals	Positive Emphasis	Negative Emphasis	Recent Demands
Way others see you	**0.75**	0.26	0.00	0.04	0.13	0.13
People your age	**0.75**	0.18	-0.01	0.02	0.13	0.04
Life working for	**0.72**	0.29	-0.05	0.07	-0.09	0.16
Healthy others	**0.72**	0.13	0.27	-0.01	0.13	0.03
Perfect health	**0.71**	0.17	0.28	-0.03	-0.04	0.08
Others with same condition	**0.64**	0.03	0.26	0.17	0.29	0.07
Family treated for same health condition	**0.62**	0.01	0.30	0.14	0.19	0.10
Doctor said	**0.57**	0.04	**0.50**	0.08	0.09	0.13
Time before health condition	**0.55**	0.14	**0.52**	-0.06	0.04	0.06
Get out of rut	0.22	**0.77**	0.10	-0.10	0.07	0.19
Feel settled	0.27	**0.71**	0.08	-0.01	0.07	0.15
Improve mood	0.20	**0.69**	0.15	-0.24	0.18	0.21
Resolve practical problems	0.08	**0.65**	0.09	-0.03	0.21	0.14
Reduce help	0.17	**0.55**	**0.44**	0.07	0.08	0.01
Help with health	0.13	**0.53**	**0.54**	-0.03	0.05	0.05
Focus on health	0.20	0.17	**0.74**	0.07	-0.03	0.10
Recent flare-ups	0.25	0.19	**0.58**	-0.11	0.28	0.25
Things better	0.09	-0.02	0.00	**0.76**	0.08	-0.05
Keep up mood	0.00	-0.18	-0.03	**0.75**	-0.21	0.06
Emphasize positive	0.17	-0.11	0.04	**0.67**	-0.33	0.04
Gotten used to	0.04	0.11	0.04	**0.58**	**0.48**	-0.19
Determined by others	0.17	0.11	-0.01	-0.01	**0.65**	0.26
Negatives more important	0.15	0.23	0.13	-0.29	**0.64**	0.21
Worst moments	0.29	0.15	0.40	-0.22	**0.50**	0.24
Ups and downs	0.12	0.35	0.07	-0.16	**0.41**	0.38
Recent events	0.15	0.18	0.11	-0.10	0.12	**0.77**
Recent changes	0.15	0.20	0.12	0.02	0.12	**0.77**
Obligations not accomplishments	0.07	0.19	0.11	0.09	0.33	**0.48**
Eigenvalue	4.65	3.28	2.47	2.27	2.15	2.06
% Variance Explained	16.61	11.70	8.84	8.10	7.68	7.35
Cumulative Variance Explained						60.28%

Note: Bolded numbers have loadings of 0.40 or greater.

Supplemental Table 5. Summary of Key MANCOVA Results†

Model	Dependent Variables	Key Independent Variables	Multivariate Effects: Wilk's Lambda	Multivariate Effects: p	Univariate Effects: F	Univariate Effects: p	Univariate Effects: Partial Eta Squared
1							
		Cancer vs. Non-Cancer	0.99	0.065			
	QOL Composite				0.09	0.768	0.000
	Cognitive Functioning Problems				4.06	**0.044**	0.004
		Comorbidity Group	0.95	**0.000**			
	QOL Composite				15.29	**0.000**	0.048
	Cognitive Functioning Problems				5.30	**0.001**	0.017
		Cancer vs. Non-Cancer * Comorbidity Group	0.98	**0.006**			
	QOL Composite				4.96	**0.002**	0.016
	Cognitive Functioning Problems				3.21	**0.023**	0.011
2							
		Cancer vs. Non-Cancer	0.94	**0.000**			
	COVID Risk/Hardship/Conflict				3.82	0.051	0.004
	COVID Growth/Support				14.81	**0.000**	0.016
	COVID Protect/Distress				1.88	0.171	0.002
	COVID Altruism/Coping				18.27	**0.000**	0.020
		Comorbidity Group	0.98	0.232			
	COVID Risk/Hardship/Conflict				0.93	0.426	0.003
	COVID Growth/Support				1.20	0.307	0.004
	COVID Protect/Distress				2.49	0.059	0.008
	COVID Altruism/Coping				0.56	0.643	0.002
		Cancer vs. Non-Cancer * Comorbidity Group	0.97	**0.002**			
	COVID Risk/Hardship/Conflict				1.24	0.294	0.004
	COVID Growth/Support				0.13	0.944	0.000
	COVID Protect/Distress				4.21	**0.006**	0.014
	COVID Altruism/Coping				5.38	**0.001**	0.018
3							
		Cancer vs. Non-Cancer	0.98	**0.003**			
	Focused on Comparisons				3.55	0.060	0.004
	Problem Goals				0.36	0.550	0.000
	Health Goals				0.28	0.599	0.000
	Positive Emphasis				0.38	0.535	0.000
	Negative Emphasis				12.64	**0.000**	**0.013**
	Recent Demands				1.38	0.240	0.001
		Comorbidity Group	0.95	**0.000**			
	Focused on Comparisons				0.56	0.642	0.002
	Problem Goals				3.54	**0.014**	0.011
	Health Goals				5.40	**0.001**	0.017
	Positive Emphasis				1.76	0.154	0.006
	Negative Emphasis				1.00	0.392	0.003
	Recent Demands				1.51	0.209	0.005
		Cancer vs. Non-Cancer * Comorbidity Group	0.97	0.084			
	Focused on Comparisons				1.47	0.221	0.005
	Problem Goals				3.34	0.019	0.011
	Health Goals				0.93	0.428	0.003
	Positive Emphasis				0.93	0.426	0.003
	Negative Emphasis				0.12	0.950	0.000
	Recent Demands				1.49	0.217	0.005

† For full output, see Supplemental Tables 5-7

Supplemental Table 6: Full MANCOVA output for QOL Composite and Cognitive Functioning Problems

General Linear Model

Multivariate Tests[a]

Effect		Value	F	Hypothesis df	Error df	Sig.	Partial Eta Squared
Intercept	Pillai's Trace	0.019	8.627[b]	2.000	905.000	**0.000**	0.019
	Wilks' Lambda	0.981	8.627[b]	2.000	905.000	**0.000**	0.019
	Hotelling's Trace	0.019	8.627[b]	2.000	905.000	**0.000**	0.019
	Roy's Largest Root	0.019	8.627[b]	2.000	905.000	**0.000**	0.019
gender	Pillai's Trace	0.004	1.791[b]	2.000	905.000	0.167	0.004
	Wilks' Lambda	0.996	1.791[b]	2.000	905.000	0.167	0.004
	Hotelling's Trace	0.004	1.791[b]	2.000	905.000	0.167	0.004
	Roy's Largest Root	0.004	1.791[b]	2.000	905.000	0.167	0.004
white	Pillai's Trace	0.004	1.888[b]	2.000	905.000	0.152	0.004
	Wilks' Lambda	0.996	1.888[b]	2.000	905.000	0.152	0.004
	Hotelling's Trace	0.004	1.888[b]	2.000	905.000	0.152	0.004
	Roy's Largest Root	0.004	1.888[b]	2.000	905.000	0.152	0.004
Education Level	Pillai's Trace	0.023	10.805[b]	2.000	905.000	**0.000**	0.023
	Wilks' Lambda	0.977	10.805[b]	2.000	905.000	**0.000**	0.023
	Hotelling's Trace	0.024	10.805[b]	2.000	905.000	**0.000**	0.023
	Roy's Largest Root	0.024	10.805[b]	2.000	905.000	**0.000**	0.023
bills	Pillai's Trace	0.206	117.267[b]	2.000	905.000	**0.000**	0.206
	Wilks' Lambda	0.794	117.267[b]	2.000	905.000	**0.000**	0.206
	Hotelling's Trace	0.259	117.267[b]	2.000	905.000	**0.000**	0.206
	Roy's Largest Root	0.259	117.267[b]	2.000	905.000	**0.000**	0.206
live.spouse	Pillai's Trace	0.019	8.902[b]	2.000	905.000	**0.000**	0.019
	Wilks' Lambda	0.981	8.902[b]	2.000	905.000	**0.000**	0.019
	Hotelling's Trace	0.020	8.902[b]	2.000	905.000	**0.000**	0.019
	Roy's Largest Root	0.020	8.902[b]	2.000	905.000	**0.000**	0.019
live.alone	Pillai's Trace	0.006	2.881[b]	2.000	905.000	0.057	0.006
	Wilks' Lambda	0.994	2.881[b]	2.000	905.000	0.057	0.006
	Hotelling's Trace	0.006	2.881[b]	2.000	905.000	0.057	0.006
	Roy's Largest Root	0.006	2.881[b]	2.000	905.000	0.057	0.006
cancer_vs_ipsos_r	Pillai's Trace	0.006	2.744[b]	2.000	905.000	0.065	0.006
	Wilks' Lambda	0.994	2.744[b]	2.000	905.000	0.065	0.006
	Hotelling's Trace	0.006	2.744[b]	2.000	905.000	0.065	0.006
	Roy's Largest Root	0.006	2.744[b]	2.000	905.000	0.065	0.006
N_comorbid_4groups	Pillai's Trace	0.055	8.517	6.000	1812.000	**0.000**	0.027
	Wilks' Lambda	0.945	8.583[b]	6.000	1810.000	**0.000**	0.028
	Hotelling's Trace	0.057	8.648	6.000	1808.000	**0.000**	0.028
	Roy's Largest Root	0.051	15.475[c]	3.000	906.000	**0.000**	0.049
cancer_vs_ipsos_r * N_comorbid_4groups	Pillai's Trace	0.020	3.044	6.000	1812.000	**0.006**	0.010
	Wilks' Lambda	0.980	3.052[b]	6.000	1810.000	**0.006**	0.010
	Hotelling's Trace	0.020	3.061	6.000	1808.000	**0.006**	0.010
	Roy's Largest Root	0.019	5.780[c]	3.000	906.000	**0.001**	0.019

a. Design: Intercept + gender + white + Education Level + bills + live.spouse + live.alone + cancer_vs_ipsos_r + N_comorbid_4groups + cancer_vs_ipsos_r * N_comorbid_4groups
b. Exact statistic
c. The statistic is an upper bound on F that yields a lower bound on the significance level.

Tests of Between-Subjects Effects

Source		Type III Sum of Squares	df	Mean Square	F	Sig.	Partial Eta Squared
Corrected Model	QOL Composite	198.462[a]	13	15.266	19.317	**0.000**	0.217
	Cognitive Functioning Problems	229.535[b]	13	17.657	22.933	**0.000**	0.248
Intercept	QOL Composite	1.862	1	1.862	2.356	0.125	0.003
	Cognitive Functioning Problems	13.285	1	13.285	17.255	**0.000**	0.019
gender	QOL Composite	0.008	1	0.008	0.011	0.917	0.000
	Cognitive Functioning Problems	2.426	1	2.426	3.151	0.076	0.003
white	QOL Composite	0.016	1	0.016	0.021	0.886	0.000
	Cognitive Functioning Problems	2.280	1	2.280	2.961	0.086	0.003
Education Level	QOL Composite	1.315	1	1.315	1.663	0.197	0.002
	Cognitive Functioning Problems	9.882	1	9.882	12.835	**0.000**	0.014
bills	QOL Composite	92.943	1	92.943	117.604	**0.000**	0.115
	Cognitive Functioning Problems	156.429	1	156.429	203.179	**0.000**	0.183
live.spouse	QOL Composite	10.258	1	10.258	12.980	**0.000**	0.014
	Cognitive Functioning Problems	0.259	1	0.259	0.337	0.562	0.000
live.alone	QOL Composite	3.879	1	3.879	4.908	**0.027**	0.005
	Cognitive Functioning Problems	2.318	1	2.318	3.010	0.083	0.003
cancer_vs_ipsos_r	QOL Composite	0.069	1	0.069	0.087	0.768	0.000
	Cognitive Functioning Problems	3.124	1	3.124	4.058	**0.044**	0.004
N_comorbid_4groups	QOL Composite	36.255	3	12.085	15.292	**0.000**	0.048
	Cognitive Functioning Problems	12.245	3	4.082	5.301	**0.001**	0.017
cancer_vs_ipsos_r * N_comorbid_4groups	QOL Composite	11.748	3	3.916	4.955	**0.002**	0.016
	Cognitive Functioning Problems	7.409	3	2.470	3.208	**0.023**	0.011
Error	QOL Composite	716.011	906	0.790			
	Cognitive Functioning Problems	697.538	906	0.770			
Total	QOL Composite	914.513	920				
	Cognitive Functioning Problems	952.491	920				
Corrected Total	QOL Composite	914.473	919				
	Cognitive Functioning Problems	927.072	919				

a. R Squared = .217 (Adjusted R Squared = .206)

b. R Squared = .248 (Adjusted R Squared = .237)

Cancer_vs_ipsos_r * Comorbidities other than cancer

Estimates

Dependent Variable			Mean	Std. Error	95% Confidence Interval	
					Lower Bound	Upper Bound
QOL Composite	.00	.00	.208[a]	0.087	0.036	0.379
		1.00	.204[a]	0.088	0.031	0.376
		2.00	.095[a]	0.105	-0.111	0.301
		3.00	-.054[a]	0.085	-0.222	0.113
	1.00	.00	.626[a]	0.120	0.390	0.861
		1.00	.058[a]	0.098	-0.135	0.251
		2.00	-.018[a]	0.090	-0.195	0.159
		3.00	-.300[a]	0.057	-0.411	-0.188
Cognitive Functioning Problems	.00	.00	-.179[a]	0.086	-0.349	-0.010
		1.00	-.036[a]	0.087	-0.206	0.135
		2.00	-.251[a]	0.104	-0.454	-0.048
		3.00	-.115[a]	0.084	-0.281	0.050
	1.00	.00	-.646[a]	0.118	-0.879	-0.414
		1.00	-.194[a]	0.097	-0.385	-0.004
		2.00	-.260[a]	0.089	-0.435	-0.085
		3.00	-.064[a]	0.056	-0.174	0.047

a. Covariates appearing in the model are evaluated at the following values: Gender = 1.71, white White (Check all that apply) = .89, Education Level = 2.6815, Difficulty Paying Bills = 1.84, Live with Spouse/Partner = .64, live.alone Alone = .17.

Pairwise Comparisons

Dependent Variable				Mean Difference (I-J)	Std. Error	Sig.[b]	95% Confidence Interval for Difference[b] Lower Bound	Upper Bound
QOL Composite	.00	.00	1.00	-.418*	0.151	**0.006**	-0.714	-0.121
		1.00	.00	.418*	0.151	**0.006**	0.121	0.714
	1.00	.00	1.00	0.146	0.134	0.277	-0.117	0.409
		1.00	.00	-0.146	0.134	0.277	-0.409	0.117
	2.00	.00	1.00	0.113	0.140	0.418	-0.161	0.388
		1.00	.00	-0.113	0.140	0.418	-0.388	0.161
	3.00	.00	1.00	.245*	0.104	**0.019**	0.041	0.450
		1.00	.00	-.245*	0.104	**0.019**	-0.450	-0.041
Cognitive Functioning Problems	.00	.00	1.00	.467*	0.149	**0.002**	0.174	0.760
		1.00	.00	-.467*	0.149	**0.002**	-0.760	-0.174
	1.00	.00	1.00	0.159	0.132	0.230	-0.100	0.418
		1.00	.00	-0.159	0.132	0.230	-0.418	0.100
	2.00	.00	1.00	0.009	0.138	0.948	-0.262	0.280
		1.00	.00	-0.009	0.138	0.948	-0.280	0.262
	3.00	.00	1.00	-0.052	0.103	0.614	-0.253	0.150
		1.00	.00	0.052	0.103	0.614	-0.150	0.253

Based on estimated marginal means
*. The mean difference is significant at the .05 level.
b. Adjustment for multiple comparisons: Least Significant Difference (equivalent to no adjustments).

Multivariate Tests

Comorbidities other than cancer		Value	F	Hypothesis df	Error df	Sig.	Partial Eta Squared
.00	Pillai's trace	0.014	6.256[a]	2.000	905.000	**0.002**	0.014
	Wilks' lambda	0.986	6.256[a]	2.000	905.000	**0.002**	0.014
	Hotelling's trace	0.014	6.256[a]	2.000	905.000	**0.002**	0.014
	Roy's largest root	0.014	6.256[a]	2.000	905.000	**0.002**	0.014
1.00	Pillai's trace	0.005	2.183[a]	2.000	905.000	0.113	0.005
	Wilks' lambda	0.995	2.183[a]	2.000	905.000	0.113	0.005
	Hotelling's trace	0.005	2.183[a]	2.000	905.000	0.113	0.005
	Roy's largest root	0.005	2.183[a]	2.000	905.000	0.113	0.005
2.00	Pillai's trace	0.001	.417[a]	2.000	905.000	0.659	0.001
	Wilks' lambda	0.999	.417[a]	2.000	905.000	0.659	0.001
	Hotelling's trace	0.001	.417[a]	2.000	905.000	0.659	0.001
	Roy's largest root	0.001	.417[a]	2.000	905.000	0.659	0.001
3.00	Pillai's trace	0.006	2.883[a]	2.000	905.000	0.056	0.006
	Wilks' lambda	0.994	2.883[a]	2.000	905.000	0.056	0.006
	Hotelling's trace	0.006	2.883[a]	2.000	905.000	0.056	0.006
	Roy's largest root	0.006	2.883[a]	2.000	905.000	0.056	0.006

Each F tests the multivariate simple effects of cancer_vs_ipsos_r within each level combination of the other effects shown. These tests are based on the linearly independent pairwise comparisons among the estimated marginal means.

a. Exact statistic

Univariate Tests

Dependent Variable			Sum of Squares	df	Mean Square	F	Sig.	Partial Eta Squared
QOL Composite	.00	Contrast	6.039	1	6.039	7.641	**0.006**	0.008
		Error	716.011	906	0.790			
	1.00	Contrast	0.936	1	0.936	1.185	0.277	0.001
		Error	716.011	906	0.790			
	2.00	Contrast	0.518	1	0.518	0.656	0.418	0.001
		Error	716.011	906	0.790			
	3.00	Contrast	4.383	1	4.383	5.547	**0.019**	0.006
		Error	716.011	906	0.790			
Cognitive Functioning Problems	.00	Contrast	7.541	1	7.541	9.795	**0.002**	0.011
		Error	697.538	906	0.770			
	1.00	Contrast	1.113	1	1.113	1.446	0.230	0.002
		Error	697.538	906	0.770			
	2.00	Contrast	0.003	1	0.003	0.004	0.948	0.000
		Error	697.538	906	0.770			
	3.00	Contrast	0.196	1	0.196	0.254	0.614	0.000
		Error	697.538	906	0.770			

Each F tests the simple effects of cancer_vs_ipsos_r within each level combination of the other effects shown. These tests are based on the linearly independent pairwise comparisons among the estimated marginal means.

Supplemental Table 7: Full MANCOVA Output for COVID-Specific Composites

General Linear Model

Multivariate Tests[a]

Effect		Value	F	Hypothesis df	Error df	Sig.	Partial Eta Squared
Intercept	Pillai's Trace	0.025	5.793[b]	4.000	894.000	**0.000**	0.025
	Wilks' Lambda	0.975	5.793[b]	4.000	894.000	**0.000**	0.025
	Hotelling's Trace	0.026	5.793[b]	4.000	894.000	**0.000**	0.025
	Roy's Largest Root	0.026	5.793[b]	4.000	894.000	**0.000**	0.025
gender	Pillai's Trace	0.020	4.569[b]	4.000	894.000	**0.001**	0.020
	Wilks' Lambda	0.980	4.569[b]	4.000	894.000	**0.001**	0.020
	Hotelling's Trace	0.020	4.569[b]	4.000	894.000	**0.001**	0.020
	Roy's Largest Root	0.020	4.569[b]	4.000	894.000	**0.001**	0.020
white	Pillai's Trace	0.012	2.736[b]	4.000	894.000	**0.028**	0.012
	Wilks' Lambda	0.988	2.736[b]	4.000	894.000	**0.028**	0.012
	Hotelling's Trace	0.012	2.736[b]	4.000	894.000	**0.028**	0.012
	Roy's Largest Root	0.012	2.736[b]	4.000	894.000	**0.028**	0.012
Education Level	Pillai's Trace	0.050	11.667[b]	4.000	894.000	**0.000**	0.050
	Wilks' Lambda	0.950	11.667[b]	4.000	894.000	**0.000**	0.050
	Hotelling's Trace	0.052	11.667[b]	4.000	894.000	**0.000**	0.050
	Roy's Largest Root	0.052	11.667[b]	4.000	894.000	**0.000**	0.050
bills	Pillai's Trace	0.357	123.821[b]	4.000	894.000	**0.000**	0.357
	Wilks' Lambda	0.643	123.821[b]	4.000	894.000	**0.000**	0.357
	Hotelling's Trace	0.554	123.821[b]	4.000	894.000	**0.000**	0.357
	Roy's Largest Root	0.554	123.821[b]	4.000	894.000	**0.000**	0.357
live.spouse	Pillai's Trace	0.034	7.811[b]	4.000	894.000	**0.000**	0.034
	Wilks' Lambda	0.966	7.811[b]	4.000	894.000	**0.000**	0.034
	Hotelling's Trace	0.035	7.811[b]	4.000	894.000	**0.000**	0.034
	Roy's Largest Root	0.035	7.811[b]	4.000	894.000	**0.000**	0.034
live.alone	Pillai's Trace	0.011	2.447[b]	4.000	894.000	**0.045**	0.011
	Wilks' Lambda	0.989	2.447[b]	4.000	894.000	**0.045**	0.011
	Hotelling's Trace	0.011	2.447[b]	4.000	894.000	**0.045**	0.011
	Roy's Largest Root	0.011	2.447[b]	4.000	894.000	**0.045**	0.011
cancer_vs_ipsos_r	Pillai's Trace	0.061	14.613[b]	4.000	894.000	**0.000**	0.061
	Wilks' Lambda	0.939	14.613[b]	4.000	894.000	**0.000**	0.061
	Hotelling's Trace	0.065	14.613[b]	4.000	894.000	**0.000**	0.061
	Roy's Largest Root	0.065	14.613[b]	4.000	894.000	**0.000**	0.061
N_comorbid_4groups	Pillai's Trace	0.017	1.265	12.000	2688.000	0.232	0.006

	Wilks' Lambda	0.983	1.267	12.000	2365.593	0.232	0.006
	Hotelling's Trace	0.017	1.268	12.000	2678.000	0.231	0.006
	Roy's Largest Root	0.013	2.987[c]	4.000	896.000	**0.018**	0.013
cancer_vs_ipsos_r * N_comorbid_4groups	Pillai's Trace	0.034	2.579	12.000	2688.000	**0.002**	0.011
	Wilks' Lambda	0.966	2.595	12.000	2365.593	**0.002**	0.011
	Hotelling's Trace	0.035	2.609	12.000	2678.000	**0.002**	0.012
	Roy's Largest Root	0.031	6.859[c]	4.000	896.000	**0.000**	0.030

a. Design: Intercept + gender + white + Education Level + bills + live.spouse + live.alone + cancer_vs_ipsos_r + N_comorbid_4groups + cancer_vs_ipsos_r * N_comorbid_4groups

b. Exact statistic

c. The statistic is an upper bound on F that yields a lower bound on the significance level.

Tests of Between-Subjects Effects

Source		Type III Sum of S	df	Mean Square	F	Sig.	Partial Eta Squared
Corrected Model	COVID Risk/Hardship/Conflict	339.741[a]	13	26.134	40.463	**0.000**	0.370
	COVID Growth/Support	79.253[b]	13	6.096	6.643	**0.000**	0.088
	COVID Protect/Distress	124.848[c]	13	9.604	10.999	**0.000**	0.137
	COVID Altruism/Coping	173.561[d]	13	13.351	16.417	**0.000**	0.192
Intercept	COVID Risk/Hardship/Conflict	3.490	1	3.490	5.403	**0.020**	0.006
	COVID Growth/Support	0.131	1	0.131	0.143	0.706	0.000
	COVID Protect/Distress	13.520	1	13.520	15.484	**0.000**	0.017
	COVID Altruism/Coping	3.064	1	3.064	3.768	0.053	0.004
gender	COVID Risk/Hardship/Conflict	8.586	1	8.586	13.294	**0.000**	0.015
	COVID Growth/Support	0.182	1	0.182	0.199	0.656	0.000
	COVID Protect/Distress	2.696	1	2.696	3.088	0.079	0.003
	COVID Altruism/Coping	0.162	1	0.162	0.199	0.656	0.000
white	COVID Risk/Hardship/Conflict	0.823	1	0.823	1.274	0.259	0.001
	COVID Growth/Support	1.798	1	1.798	1.960	0.162	0.002
	COVID Protect/Distress	1.824	1	1.824	2.089	0.149	0.002
	COVID Altruism/Coping	2.512	1	2.512	3.089	0.079	0.003
Education Level	COVID Risk/Hardship/Conflict	1.422	1	1.422	2.202	0.138	0.002
	COVID Growth/Support	0.355	1	0.355	0.387	0.534	0.000
	COVID Protect/Distress	3.263	1	3.263	3.737	0.054	0.004
	COVID Altruism/Coping	31.214	1	31.214	38.382	**0.000**	0.041
bills	COVID Risk/Hardship/Conflict	251.337	1	251.337	389.143	**0.000**	0.303
	COVID Growth/Support	6.531	1	6.531	7.116	**0.008**	0.008
	COVID Protect/Distress	84.271	1	84.271	96.516	**0.000**	0.097
	COVID Altruism/Coping	33.930	1	33.930	41.721	**0.000**	0.044
live.spouse	COVID Risk/Hardship/Conflict	0.003	1	0.003	0.005	0.945	0.000
	COVID Growth/Support	26.125	1	26.125	28.468	**0.000**	0.031
	COVID Protect/Distress	0.035	1	0.035	0.040	0.841	0.000
	COVID Altruism/Coping	8.777	1	8.777	10.792	**0.001**	0.012
live.alone	COVID Risk/Hardship/Conflict	5.882	1	5.882	9.106	**0.003**	0.010
	COVID Growth/Support	0.123	1	0.123	0.134	0.715	0.000
	COVID Protect/Distress	0.001	1	0.001	0.001	0.972	0.000

	COVID Altruism/Coping	0.301	1	0.301	0.370	0.543	0.000
cancer_vs_ipsos_r	COVID Risk/Hardship/Conflict	2.468	1	2.468	3.822	0.051	0.004
	COVID Growth/Support	13.590	1	13.590	14.809	**0.000**	0.016
	COVID Protect/Distress	1.639	1	1.639	1.878	0.171	0.002
	COVID Altruism/Coping	14.862	1	14.862	18.274	**0.000**	0.020
N_comorbid_4groups	COVID Risk/Hardship/Conflict	1.800	3	0.600	0.929	0.426	0.003
	COVID Growth/Support	3.313	3	1.104	1.203	0.307	0.004
	COVID Protect/Distress	6.511	3	2.170	2.486	0.059	0.008
	COVID Altruism/Coping	1.360	3	0.453	0.557	0.643	0.002
cancer_vs_ipsos_r * N_comorbid_4groups	COVID Risk/Hardship/Conflict	2.401	3	0.800	1.239	0.294	0.004
	COVID Growth/Support	0.352	3	0.117	0.128	0.944	0.000
	COVID Protect/Distress	11.032	3	3.677	4.212	**0.006**	0.014
	COVID Altruism/Coping	13.119	3	4.373	5.377	**0.001**	0.018
Error	COVID Risk/Hardship/Conflict	579.348	897	0.646			
	COVID Growth/Support	823.183	897	0.918			
	COVID Protect/Distress	783.192	897	0.873			
	COVID Altruism/Coping	729.494	897	0.813			
Total	COVID Risk/Hardship/Conflict	919.102	911				
	COVID Growth/Support	902.503	911				
	COVID Protect/Distress	908.043	911				
	COVID Altruism/Coping	903.193	911				
Corrected Total	COVID Risk/Hardship/Conflict	919.089	910				
	COVID Growth/Support	902.436	910				
	COVID Protect/Distress	908.041	910				
	COVID Altruism/Coping	903.055	910				

a. R Squared = .370 (Adjusted R Squared = .361)
b. R Squared = .088 (Adjusted R Squared = .075)
c. R Squared = .137 (Adjusted R Squared = .125)
d. R Squared = .192 (Adjusted R Squared = .180)

Cancer_vs_ipsos_r * Comorbidities other than cancer

Estimates

Dependent Variable			Mean	Std. Error	95% Confidence Interval	
					Lower Bound	Upper Bound
COVID Risk/Hardship/Conflict	.00	.00	.130[a]	0.079	-0.026	0.286
		1.00	.189[a]	0.080	0.031	0.347
		2.00	-.040[a]	0.094	-0.225	0.145
		3.00	-.023[a]	0.079	-0.177	0.132
	1.00	.00	-.113[a]	0.109	-0.328	0.101
		1.00	-.042[a]	0.089	-0.217	0.132
		2.00	-.079[a]	0.081	-0.239	0.081
		3.00	-.029[a]	0.052	-0.130	0.072

COVID Growth/Support	.00	.00	.269[a]	0.095	0.083	0.454
		1.00	.224[a]	0.096	0.036	0.412
		2.00	.158[a]	0.112	-0.062	0.379
		3.00	.154[a]	0.094	-0.030	0.338
	1.00	.00	.018[a]	0.131	-0.238	0.274
		1.00	-.071[a]	0.106	-0.278	0.137
		2.00	-.154[a]	0.097	-0.345	0.036
		3.00	-.209[a]	0.061	-0.329	-0.088
COVID Protect/Distress	.00	.00	-.027[a]	0.092	-0.208	0.155
		1.00	.022[a]	0.093	-0.161	0.205
		2.00	.087[a]	0.110	-0.128	0.302
		3.00	-.064[a]	0.091	-0.244	0.115
	1.00	.00	-.387[a]	0.127	-0.637	-0.137
		1.00	-.037[a]	0.103	-0.239	0.166
		2.00	-.148[a]	0.095	-0.333	0.038
		3.00	.165[a]	0.060	0.047	0.282
COVID Altruism/Coping	.00	.00	-.304[a]	0.089	-0.479	-0.129
		1.00	-.010[a]	0.090	-0.187	0.167
		2.00	-.133[a]	0.106	-0.340	0.075
		3.00	-.029[a]	0.088	-0.202	0.145
	1.00	.00	.454[a]	0.123	0.213	0.695
		1.00	.184[a]	0.100	-0.011	0.380
		2.00	.151[a]	0.091	-0.029	0.330
		3.00	.013[a]	0.058	-0.101	0.126

a. Covariates appearing in the model are evaluated at the following values: Gender = 1.72, white White (Check all that apply) = .89, Education Level = 2.6839, Difficulty Paying Bills = 1.84, Live with Spouse/Partner = .64, live.alone Alone = .17.

Pairwise Comparisons

Dependent Variable				Mean Difference (I-J)	Std. Error	Sig.[b]	95% Confidence Interval for Difference[b]	
							Lower Bound	Upper Bound
COVID Risk/Hardship/Conflict	.00	.00	1.00	0.243	0.138	0.078	-0.027	0.514
		1.00	.00	-0.243	0.138	0.078	-0.514	0.027
	1.00	.00	1.00	0.232	0.122	0.057	-0.007	0.470
		1.00	.00	-0.232	0.122	0.057	-0.470	0.007
	2.00	.00	1.00	0.039	0.126	0.756	-0.208	0.286
		1.00	.00	-0.039	0.126	0.756	-0.286	0.208
	3.00	.00	1.00	0.006	0.095	0.946	-0.181	0.193
		1.00	.00	-0.006	0.095	0.946	-0.193	0.181
COVID Growth/Support	.00	.00	1.00	0.251	0.164	0.128	-0.072	0.573

		1.00	.00	-0.251	0.164	0.128	-0.573	0.072
	1.00	.00	1.00	.295[*]	0.145	**0.042**	0.011	0.580
		1.00	.00	-.295[*]	0.145	**0.042**	-0.580	-0.011
	2.00	.00	1.00	.313[*]	0.150	**0.038**	0.018	0.607
		1.00	.00	-.313[*]	0.150	**0.038**	-0.607	-0.018
	3.00	.00	1.00	.363[*]	0.114	**0.001**	0.140	0.586
		1.00	.00	-.363[*]	0.114	**0.001**	-0.586	-0.140
COVID Protect/Distress	.00	.00	1.00	.360[*]	0.160	**0.025**	0.046	0.674
		1.00	.00	-.360[*]	0.160	**0.025**	-0.674	-0.046
	1.00	.00	1.00	0.059	0.141	0.677	-0.219	0.336
		1.00	.00	-0.059	0.141	0.677	-0.336	0.219
	2.00	.00	1.00	0.234	0.146	0.110	-0.053	0.522
		1.00	.00	-0.234	0.146	0.110	-0.522	0.053
	3.00	.00	1.00	-.229[*]	0.111	**0.039**	-0.447	-0.012
		1.00	.00	.229[*]	0.111	**0.039**	0.012	0.447
COVID Altruism/Coping	.00	.00	1.00	-.758[*]	0.155	**0.000**	-1.062	-0.455
		1.00	.00	.758[*]	0.155	**0.000**	0.455	1.062
	1.00	.00	1.00	-0.195	0.136	0.154	-0.462	0.073
		1.00	.00	0.195	0.136	0.154	-0.073	0.462
	2.00	.00	1.00	-.283[*]	0.141	**0.045**	-0.561	-0.006
		1.00	.00	.283[*]	0.141	**0.045**	0.006	0.561
	3.00	.00	1.00	-0.041	0.107	0.701	-0.251	0.169
		1.00	.00	0.041	0.107	0.701	-0.169	0.251

Based on estimated marginal means

*. The mean difference is significant at the .05 level.

b. Adjustment for multiple comparisons: Least Significant Difference (equivalent to no adjustments).

Multivariate Tests

Comorbidities other than cancer		Value	F	Hypothesis df	Error df	Sig.	artial Eta Squared
.00	Pillai's trace	0.047	10.912[a]	4.000	894.000	**0.000**	0.047
	Wilks' lambda	0.953	10.912[a]	4.000	894.000	**0.000**	0.047
	Hotelling's trace	0.049	10.912[a]	4.000	894.000	**0.000**	0.047
	Roy's largest root	0.049	10.912[a]	4.000	894.000	**0.000**	0.047
1.00	Pillai's trace	0.016	3.679[a]	4.000	894.000	**0.006**	0.016
	Wilks' lambda	0.984	3.679[a]	4.000	894.000	**0.006**	0.016
	Hotelling's trace	0.016	3.679[a]	4.000	894.000	**0.006**	0.016
	Roy's largest root	0.016	3.679[a]	4.000	894.000	**0.006**	0.016
2.00	Pillai's trace	0.016	3.689[a]	4.000	894.000	**0.005**	0.016
	Wilks' lambda	0.984	3.689[a]	4.000	894.000	**0.005**	0.016
	Hotelling's trace	0.017	3.689[a]	4.000	894.000	**0.005**	0.016
	Roy's largest root	0.017	3.689[a]	4.000	894.000	**0.005**	0.016

3.00	Pillai's trace	0.020	4.550[a]	4.000	894.000	**0.001**	0.020
	Wilks' lambda	0.980	4.550[a]	4.000	894.000	**0.001**	0.020
	Hotelling's trace	0.020	4.550[a]	4.000	894.000	**0.001**	0.020
	Roy's largest root	0.020	4.550[a]	4.000	894.000	**0.001**	0.020

Each F tests the multivariate simple effects of cancer_vs_ipsos_r within each level combination of the other effects shown. These tests are based on the linearly independent pairwise comparisons among the estimated marginal means.

a. Exact statistic

Univariate Tests

Dependent Variable			Sum of Squares	df	Mean Square	F	Sig.	Partial Eta Squared
COVID Risk/Hardship/Conflict	.00	Contrast	2.016	1	2.016	3.121	0.078	0.003
		Error	579.348	897	0.646			
	1.00	Contrast	2.342	1	2.342	3.627	0.057	0.004
		Error	579.348	897	0.646			
	2.00	Contrast	0.062	1	0.062	0.096	0.756	0.000
		Error	579.348	897	0.646			
	3.00	Contrast	0.003	1	0.003	0.005	0.946	0.000
		Error	579.348	897	0.646			
COVID Growth/Support	.00	Contrast	2.135	1	2.135	2.327	0.128	0.003
		Error	823.183	897	0.918			
	1.00	Contrast	3.803	1	3.803	4.144	**0.042**	0.005
		Error	823.183	897	0.918			
	2.00	Contrast	3.979	1	3.979	4.336	**0.038**	0.005
		Error	823.183	897	0.918			
	3.00	Contrast	9.379	1	9.379	10.220	**0.001**	0.011
		Error	823.183	897	0.918			
COVID Protect/Distress	.00	Contrast	4.407	1	4.407	5.048	**0.025**	0.006
		Error	783.192	897	0.873			
	1.00	Contrast	0.152	1	0.152	0.174	0.677	0.000
		Error	783.192	897	0.873			
	2.00	Contrast	2.237	1	2.237	2.562	0.110	0.003
		Error	783.192	897	0.873			
	3.00	Contrast	3.733	1	3.733	4.275	**0.039**	0.005
		Error	783.192	897	0.873			
COVID Altruism/Coping	.00	Contrast	19.550	1	19.550	24.039	**0.000**	0.026
		Error	729.494	897	0.813			
	1.00	Contrast	1.655	1	1.655	2.035	0.154	0.002
		Error	729.494	897	0.813			
	2.00	Contrast	3.269	1	3.269	4.020	**0.045**	0.004
		Error	729.494	897	0.813			
	3.00	Contrast	0.120	1	0.120	0.148	0.701	0.000
		Error	729.494	897	0.813			

Each F tests the simple effects of cancer_vs_ipsos_r within each level combination of the other effects shown. These tests are based on the linearly independent pairwise comparisons among the estimated marginal means.

Supplemental Table 8: Full MANCOVA Output for Appraisal Composites

General Linear Model

Multivariate Tests[a]

Effect		Value	F	Hypothesis df	Error df	Sig.	Partial Eta Squared
Intercept	Pillai's Trace	0.020	3.150[b]	6.000	921.000	**0.005**	0.020
	Wilks' Lambda	0.980	3.150[b]	6.000	921.000	**0.005**	0.020
	Hotelling's Trace	0.021	3.150[b]	6.000	921.000	**0.005**	0.020
	Roy's Largest Root	0.021	3.150[b]	6.000	921.000	**0.005**	0.020
gender	Pillai's Trace	0.029	4.552[b]	6.000	921.000	**0.000**	0.029
	Wilks' Lambda	0.971	4.552[b]	6.000	921.000	**0.000**	0.029
	Hotelling's Trace	0.030	4.552[b]	6.000	921.000	**0.000**	0.029
	Roy's Largest Root	0.030	4.552[b]	6.000	921.000	**0.000**	0.029
white	Pillai's Trace	0.012	1.860[b]	6.000	921.000	0.085	0.012
	Wilks' Lambda	0.988	1.860[b]	6.000	921.000	0.085	0.012
	Hotelling's Trace	0.012	1.860[b]	6.000	921.000	0.085	0.012
	Roy's Largest Root	0.012	1.860[b]	6.000	921.000	0.085	0.012
Education Level	Pillai's Trace	0.020	3.131[b]	6.000	921.000	**0.005**	0.020
	Wilks' Lambda	0.980	3.131[b]	6.000	921.000	**0.005**	0.020
	Hotelling's Trace	0.020	3.131[b]	6.000	921.000	**0.005**	0.020
	Roy's Largest Root	0.020	3.131[b]	6.000	921.000	**0.005**	0.020
bills	Pillai's Trace	0.218	42.897[b]	6.000	921.000	**0.000**	0.218
	Wilks' Lambda	0.782	42.897[b]	6.000	921.000	**0.000**	0.218
	Hotelling's Trace	0.279	42.897[b]	6.000	921.000	**0.000**	0.218
	Roy's Largest Root	0.279	42.897[b]	6.000	921.000	**0.000**	0.218
live.spouse	Pillai's Trace	0.023	3.560[b]	6.000	921.000	**0.002**	0.023
	Wilks' Lambda	0.977	3.560[b]	6.000	921.000	**0.002**	0.023
	Hotelling's Trace	0.023	3.560[b]	6.000	921.000	**0.002**	0.023
	Roy's Largest Root	0.023	3.560[b]	6.000	921.000	**0.002**	0.023
live.alone	Pillai's Trace	0.009	1.430[b]	6.000	921.000	0.200	0.009
	Wilks' Lambda	0.991	1.430[b]	6.000	921.000	0.200	0.009
	Hotelling's Trace	0.009	1.430[b]	6.000	921.000	0.200	0.009
	Roy's Largest Root	0.009	1.430[b]	6.000	921.000	0.200	0.009
cancer_vs_ipsos_r	Pillai's Trace	0.021	3.336[b]	6.000	921.000	**0.003**	0.021
	Wilks' Lambda	0.979	3.336[b]	6.000	921.000	**0.003**	0.021
	Hotelling's Trace	0.022	3.336[b]	6.000	921.000	**0.003**	0.021
	Roy's Largest Root	0.022	3.336[b]	6.000	921.000	**0.003**	0.021
N_comorbid_4groups	Pillai's Trace	0.048	2.479	18.000	2769.000	**0.001**	0.016
	Wilks' Lambda	0.953	2.494	18.000	2605.467	**0.000**	0.016
	Hotelling's Trace	0.049	2.508	18.000	2759.000	**0.000**	0.016

	Roy's Largest Root	0.039	5.976[c]	6.000	923.000	**0.000**	0.037
cancer_vs_ipsos_r * N_comorbid_4groups	Pillai's Trace	0.029	1.488	18.000	2769.000	0.084	0.010
	Wilks' Lambda	0.971	1.490	18.000	2605.467	0.084	0.010
	Hotelling's Trace	0.029	1.492	18.000	2759.000	0.083	0.010
	Roy's Largest Root	0.020	3.022[c]	6.000	923.000	**0.006**	0.019

a. Design: Intercept + gender + white + Education Level + bills + live.spouse + live.alone + cancer_vs_ipsos_r + N_comorbid_4groups + cancer_vs_ipsos_r * N_comorbid_4groups

b. Exact statistic

c. The statistic is an upper bound on F that yields a lower bound on the significance level.

Tests of Between-Subjects Effects

Source		Type III Sum of Squares	df	Mean Square	F	Sig.	Partial Eta Squared
Corrected Model	Focused on Comparisons	82.305[a]	13	6.331	6.883	**0.000**	0.088
	Problem Goals	150.815[b]	13	11.601	13.722	**0.000**	0.162
	Health Goals	63.545[c]	13	4.888	5.142	**0.000**	0.067
	Positive Emphasis	25.755[d]	13	1.981	2.070	**0.014**	0.028
	Negative Emphasis	94.188[e]	13	7.245	8.017	**0.000**	0.101
	Recent Demands	37.222[f]	13	2.863	2.946	**0.000**	0.040
Intercept	Focused on Comparisons	0.189	1	0.189	0.205	0.650	0.000
	Problem Goals	7.918	1	7.918	9.366	**0.002**	0.010
	Health Goals	3.245	1	3.245	3.413	0.065	0.004
	Positive Emphasis	0.548	1	0.548	0.573	0.449	0.001
	Negative Emphasis	0.129	1	0.129	0.143	0.705	0.000
	Recent Demands	4.825	1	4.825	4.965	**0.026**	0.005
gender	Focused on Comparisons	6.582	1	6.582	7.156	**0.008**	0.008
	Problem Goals	2.546	1	2.546	3.011	0.083	0.003
	Health Goals	7.941	1	7.941	8.354	**0.004**	0.009
	Positive Emphasis	1.884	1	1.884	1.968	0.161	0.002
	Negative Emphasis	4.801	1	4.801	5.313	**0.021**	0.006
	Recent Demands	0.358	1	0.358	0.368	0.544	0.000
white	Focused on Comparisons	2.469	1	2.469	2.684	0.102	0.003
	Problem Goals	1.396	1	1.396	1.651	0.199	0.002
	Health Goals	5.330	1	5.330	5.607	**0.018**	0.006
	Positive Emphasis	0.164	1	0.164	0.171	0.679	0.000
	Negative Emphasis	0.052	1	0.052	0.057	0.811	0.000
	Recent Demands	0.096	1	0.096	0.099	0.753	0.000
Education Level	Focused on Comparisons	11.428	1	11.428	12.425	**0.000**	0.013
	Problem Goals	2.903	1	2.903	3.434	0.064	0.004
	Health Goals	0.299	1	0.299	0.315	0.575	0.000
	Positive Emphasis	1.241	1	1.241	1.297	0.255	0.001
	Negative Emphasis	0.009	1	0.009	0.010	0.921	0.000
	Recent Demands	0.258	1	0.258	0.265	0.607	0.000
bills	Focused on Comparisons	26.003	1	26.003	28.271	**0.000**	0.030
	Problem Goals	84.267	1	84.267	99.673	**0.000**	0.097
	Health Goals	15.790	1	15.790	16.610	**0.000**	0.018
	Positive Emphasis	3.272	1	3.272	3.418	0.065	0.004
	Negative Emphasis	40.413	1	40.413	44.720	**0.000**	0.046
	Recent Demands	14.268	1	14.268	14.682	**0.000**	0.016

live.spouse	Focused on Comparisons	1.923	1	1.923	2.091	0.149	0.002
	Problem Goals	11.188	1	11.188	13.234	**0.000**	0.014
	Health Goals	0.183	1	0.183	0.193	0.661	0.000
	Positive Emphasis	2.991	1	2.991	3.124	0.077	0.003
	Negative Emphasis	1.431	1	1.431	1.584	0.209	0.002
	Recent Demands	1.924	1	1.924	1.980	0.160	0.002
live.alone	Focused on Comparisons	0.797	1	0.797	0.866	0.352	0.001
	Problem Goals	0.215	1	0.215	0.255	0.614	0.000
	Health Goals	2.349	1	2.349	2.472	0.116	0.003
	Positive Emphasis	1.107	1	1.107	1.157	0.282	0.001
	Negative Emphasis	2.270	1	2.270	2.512	0.113	0.003
	Recent Demands	0.322	1	0.322	0.331	0.565	0.000
cancer_vs_ipsos_r	Focused on Comparisons	3.267	1	3.267	3.552	0.060	0.004
	Problem Goals	0.302	1	0.302	0.358	0.550	0.000
	Health Goals	0.263	1	0.263	0.276	0.599	0.000
	Positive Emphasis	0.368	1	0.368	0.385	0.535	0.000
	Negative Emphasis	11.423	1	11.423	12.641	**0.000**	**0.013**
	Recent Demands	1.344	1	1.344	1.383	0.240	0.001
N_comorbid_4groups	Focused on Comparisons	1.545	3	0.515	0.560	0.642	0.002
	Problem Goals	8.981	3	2.994	3.541	**0.014**	0.011
	Health Goals	15.395	3	5.132	5.398	**0.001**	0.017
	Positive Emphasis	5.048	3	1.683	1.758	0.154	0.006
	Negative Emphasis	2.714	3	0.905	1.001	0.392	0.003
	Recent Demands	4.414	3	1.471	1.514	0.209	0.005
cancer_vs_ipsos_r * N_comorbid_4groups	Focused on Comparisons	4.060	3	1.353	1.472	0.221	0.005
	Problem Goals	8.478	3	2.826	3.343	**0.019**	0.011
	Health Goals	2.641	3	0.880	0.926	0.428	0.003
	Positive Emphasis	2.667	3	0.889	0.929	0.426	0.003
	Negative Emphasis	0.318	3	0.106	0.117	0.950	0.000
	Recent Demands	4.331	3	1.444	1.486	0.217	0.005
Error	Focused on Comparisons	851.727	926	0.920			
	Problem Goals	782.868	926	0.845			
	Health Goals	880.283	926	0.951			
	Positive Emphasis	886.474	926	0.957			
	Negative Emphasis	836.809	926	0.904			
	Recent Demands	899.855	926	0.972			
Total	Focused on Comparisons	934.203	940				
	Problem Goals	933.701	940				
	Health Goals	943.853	940				
	Positive Emphasis	912.480	940				
	Negative Emphasis	930.996	940				
	Recent Demands	937.155	940				
Corrected Total	Focused on Comparisons	934.032	939				
	Problem Goals	933.683	939				
	Health Goals	943.828	939				
	Positive Emphasis	912.229	939				
	Negative Emphasis	930.996	939				
	Recent Demands	937.078	939				

a. R Squared = .088 (Adjusted R Squared = .075)
b. R Squared = .162 (Adjusted R Squared = .150)
c. R Squared = .067 (Adjusted R Squared = .054)
d. R Squared = .028 (Adjusted R Squared = .015)
e. R Squared = .101 (Adjusted R Squared = .089)

f. R Squared = .040 (Adjusted R Squared = .026)

Cancer_vs_ipsos_r * Comorbidities other than cancer

Estimates

Dependent Variable			Mean	Std. Error	onfidence Interval	
					Lower Boun	pper Bound
Focused on Comparisons	.00	.00	.118[a]	0.092	-0.063	0.298
		1.00	.078[a]	0.094	-0.106	0.261
		2.00	.063[a]	0.111	-0.155	0.280
		3.00	.139[a]	0.092	-0.040	0.319
	1.00	.00	-.203[a]	0.128	-0.454	0.049
		1.00	.086[a]	0.105	-0.121	0.293
		2.00	.045[a]	0.096	-0.143	0.234
		3.00	-.120[a]	0.061	-0.240	0.000
Problem Goals	.00	.00	-.027[a]	0.088	-0.200	0.146
		1.00	-.058[a]	0.090	-0.234	0.118
		2.00	-.052[a]	0.106	-0.261	0.156
		3.00	-.013[a]	0.088	-0.185	0.159
	1.00	.00	-.422[a]	0.123	-0.663	-0.180
		1.00	-.024[a]	0.101	-0.223	0.174
		2.00	-.043[a]	0.092	-0.224	0.138
		3.00	.159[a]	0.059	0.044	0.274
Health Goals	.00	.00	-.166[a]	0.093	-0.349	0.018
		1.00	-.098[a]	0.095	-0.285	0.089
		2.00	-.102[a]	0.113	-0.323	0.120
		3.00	.051[a]	0.093	-0.132	0.233
	1.00	.00	-.242[a]	0.130	-0.498	0.014
		1.00	-.138[a]	0.107	-0.348	0.073
		2.00	-.012[a]	0.098	-0.203	0.180
		3.00	.243[a]	0.062	0.122	0.365
Positive Emphasis	.00	.00	.065[a]	0.094	-0.119	0.249
		1.00	.141[a]	0.095	-0.046	0.328
		2.00	.039[a]	0.113	-0.183	0.261
		3.00	.047[a]	0.093	-0.136	0.230
	1.00	.00	.213[a]	0.131	-0.044	0.470
		1.00	.100[a]	0.108	-0.112	0.311
		2.00	-.126[a]	0.098	-0.319	0.066
		3.00	-.092[a]	0.062	-0.214	0.030
Negative Emphasis	.00	.00	.161[a]	0.091	-0.018	0.339
		1.00	.097[a]	0.093	-0.085	0.279
		2.00	.242[a]	0.110	0.027	0.458
		3.00	.142[a]	0.091	-0.036	0.320

	1.00	.00	-.155[a]	0.127	-0.405	0.094
		1.00	-.141[a]	0.105	-0.347	0.064
		2.00	.010[a]	0.095	-0.177	0.197
		3.00	-.171[a]	0.061	-0.290	-0.052
Recent Demands	.00	.00	-.130[a]	0.094	-0.315	0.056
		1.00	-.013[a]	0.096	-0.202	0.176
		2.00	-.089[a]	0.114	-0.313	0.135
		3.00	-.092[a]	0.094	-0.276	0.093
	1.00	.00	-.067[a]	0.132	-0.326	0.192
		1.00	.026[a]	0.108	-0.187	0.239
		2.00	-.123[a]	0.099	-0.317	0.071
		3.00	.217[a]	0.063	0.094	0.341

a. Covariates appearing in the model are evaluated at the following values: Gender = 1.71, white White (Check all that apply) = .89, Education Level = 2.6766, Difficulty Paying Bills = 1.84, Live with Spouse/Partner = .63, live.alone Alone = .17.

Pairwise Comparisons

Dependent Variable				Mean Difference (I	Std. Error	Sig.[b]	ice Interval for Difference[b]	
							Lower Boun	Upper Bound
Focused on Comparisons	.00	.00	1.00	.320*	0.161	**0.047**	0.005	0.636
		1.00	.00	-.320*	0.161	**0.047**	-0.636	-0.005
	1.00	.00	1.00	-0.008	0.143	0.953	-0.289	0.272
		1.00	.00	0.008	0.143	0.953	-0.272	0.289
	2.00	.00	1.00	0.017	0.148	0.908	-0.274	0.308
		1.00	.00	-0.017	0.148	0.908	-0.308	0.274
	3.00	.00	1.00	.259*	0.112	**0.020**	0.040	0.479
		1.00	.00	-.259*	0.112	**0.020**	-0.479	-0.040
Problem Goals	.00	.00	1.00	.395*	0.154	**0.011**	0.092	0.697
		1.00	.00	-.395*	0.154	**0.011**	-0.697	-0.092
	1.00	.00	1.00	-0.034	0.137	0.803	-0.303	0.235
		1.00	.00	0.034	0.137	0.803	-0.235	0.303
	2.00	.00	1.00	-0.009	0.142	0.950	-0.288	0.270
		1.00	.00	0.009	0.142	0.950	-0.270	0.288
	3.00	.00	1.00	-0.173	0.107	0.107	-0.383	0.038
		1.00	.00	0.173	0.107	0.107	-0.038	0.383
Health Goals	.00	.00	1.00	0.076	0.163	0.642	-0.245	0.397
		1.00	.00	-0.076	0.163	0.642	-0.397	0.245
	1.00	.00	1.00	0.040	0.145	0.783	-0.246	0.326
		1.00	.00	-0.040	0.145	0.783	-0.326	0.246
	2.00	.00	1.00	-0.090	0.151	0.550	-0.386	0.206
		1.00	.00	0.090	0.151	0.550	-0.206	0.386
	3.00	.00	1.00	-0.193	0.113	0.090	-0.415	0.030
		1.00	.00	0.193	0.113	0.090	-0.030	0.415
Positive Emphasis	.00	.00	1.00	-0.149	0.164	0.365	-0.470	0.173
		1.00	.00	0.149	0.164	0.365	-0.173	0.470
	1.00	.00	1.00	0.042	0.146	0.776	-0.245	0.328
		1.00	.00	-0.042	0.146	0.776	-0.328	0.245
	2.00	.00	1.00	0.165	0.151	0.275	-0.132	0.462

		1.00	.00	-0.165	0.151	0.275	-0.462	0.132
	3.00	.00	1.00	0.139	0.114	0.222	-0.084	0.363
		1.00	.00	-0.139	0.114	0.222	-0.363	0.084
Negative Emphasis	.00	.00	1.00	.316[*]	0.159	**0.048**	0.003	0.628
		1.00	.00	-.316[*]	0.159	**0.048**	-0.628	-0.003
	1.00	.00	1.00	0.239	0.142	0.093	-0.040	0.517
		1.00	.00	-0.239	0.142	0.093	-0.517	0.040
	2.00	.00	1.00	0.233	0.147	0.114	-0.056	0.521
		1.00	.00	-0.233	0.147	0.114	-0.521	0.056
	3.00	.00	1.00	.313[*]	0.111	**0.005**	0.096	0.530
		1.00	.00	-.313[*]	0.111	**0.005**	-0.530	-0.096
Recent Demands	.00	.00	1.00	-0.063	0.165	0.702	-0.387	0.261
		1.00	.00	0.063	0.165	0.702	-0.261	0.387
	1.00	.00	1.00	-0.039	0.147	0.791	-0.328	0.250
		1.00	.00	0.039	0.147	0.791	-0.250	0.328
	2.00	.00	1.00	0.034	0.152	0.824	-0.265	0.333
		1.00	.00	-0.034	0.152	0.824	-0.333	0.265
	3.00	.00	1.00	-.309[*]	0.115	**0.007**	-0.534	-0.084
		1.00	.00	.309[*]	0.115	**0.007**	0.084	0.534

Based on estimated marginal means

*. The mean difference is significant at the .05 level.

b. Adjustment for multiple comparisons: Least Significant Difference (equivalent to no adjustments).

Multivariate Tests

Comorbidities other than cancer		Value	F	Hypothesis df	Error df	Sig.	tial Eta Squared
.00	Pillai's trace	0.020	3.076[a]	6.000	921.000	0.005	0.020
	Wilks' lambda	0.980	3.076[a]	6.000	921.000	0.005	0.020
	Hotelling's trace	0.020	3.076[a]	6.000	921.000	0.005	0.020
	Roy's largest root	0.020	3.076[a]	6.000	921.000	0.005	0.020
1.00	Pillai's trace	0.003	.516[a]	6.000	921.000	0.796	0.003
	Wilks' lambda	0.997	.516[a]	6.000	921.000	0.796	0.003
	Hotelling's trace	0.003	.516[a]	6.000	921.000	0.796	0.003
	Roy's largest root	0.003	.516[a]	6.000	921.000	0.796	0.003
2.00	Pillai's trace	0.004	.684[a]	6.000	921.000	0.663	0.004
	Wilks' lambda	0.996	.684[a]	6.000	921.000	0.663	0.004
	Hotelling's trace	0.004	.684[a]	6.000	921.000	0.663	0.004
	Roy's largest root	0.004	.684[a]	6.000	921.000	0.663	0.004
3.00	Pillai's trace	0.029	4.660[a]	6.000	921.000	0.000	0.029
	Wilks' lambda	0.971	4.660[a]	6.000	921.000	0.000	0.029
	Hotelling's trace	0.030	4.660[a]	6.000	921.000	0.000	0.029
	Roy's largest root	0.030	4.660[a]	6.000	921.000	0.000	0.029

Each F tests the multivariate simple effects of cancer_vs_ipsos_r within each level combination of the other effects shown. These tests are based on the linearly independent pairwise comparisons among the estimated marginal means.

a. Exact statistic

Univariate Tests

Dependent Variable			Sum of Squar	df	Mean Squar	F	Sig.	Partial Eta Square
Focused on Comparisons	.00	Contrast	3.650	1	3.650	3.968	0.047	0.004
		Error	851.727	926	0.920			
	1.00	Contrast	0.003	1	0.003	0.003	0.953	0.000
		Error	851.727	926	0.920			
	2.00	Contrast	0.012	1	0.012	0.013	0.908	0.000
		Error	851.727	926	0.920			
	3.00	Contrast	4.966	1	4.966	5.399	0.020	0.006
		Error	851.727	926	0.920			
Problem Goals	.00	Contrast	5.545	1	5.545	6.559	0.011	0.007
		Error	782.868	926	0.845			
	1.00	Contrast	0.052	1	0.052	0.062	0.803	0.000
		Error	782.868	926	0.845			
	2.00	Contrast	0.003	1	0.003	0.004	0.950	0.000
		Error	782.868	926	0.845			
	3.00	Contrast	2.197	1	2.197	2.599	0.107	0.003
		Error	782.868	926	0.845			
Health Goals	.00	Contrast	0.206	1	0.206	0.217	0.642	0.000
		Error	880.283	926	0.951			
	1.00	Contrast	0.072	1	0.072	0.076	0.783	0.000
		Error	880.283	926	0.951			
	2.00	Contrast	0.340	1	0.340	0.358	0.550	0.000
		Error	880.283	926	0.951			
	3.00	Contrast	2.740	1	2.740	2.882	0.090	0.003
		Error	880.283	926	0.951			
Positive Emphasis	.00	Contrast	0.787	1	0.787	0.822	0.365	0.001
		Error	886.474	926	0.957			
	1.00	Contrast	0.077	1	0.077	0.081	0.776	0.000
		Error	886.474	926	0.957			
	2.00	Contrast	1.144	1	1.144	1.195	0.275	0.001
		Error	886.474	926	0.957			
	3.00	Contrast	1.430	1	1.430	1.494	0.222	0.002
		Error	886.474	926	0.957			
Negative Emphasis	.00	Contrast	3.553	1	3.553	3.932	0.048	0.004
		Error	836.809	926	0.904			
	1.00	Contrast	2.556	1	2.556	2.829	0.093	0.003
		Error	836.809	926	0.904			
	2.00	Contrast	2.263	1	2.263	2.504	0.114	0.003
		Error	836.809	926	0.904			
	3.00	Contrast	7.224	1	7.224	7.993	0.005	0.009
		Error	836.809	926	0.904			
Recent Demands	.00	Contrast	0.143	1	0.143	0.147	0.702	0.000
		Error	899.855	926	0.972			
	1.00	Contrast	0.068	1	0.068	0.070	0.791	0.000
		Error	899.855	926	0.972			
	2.00	Contrast	0.048	1	0.048	0.049	0.824	0.000
		Error	899.855	926	0.972			
	3.00	Contrast	7.044	1	7.044	7.248	0.007	0.008
		Error	899.855	926	0.972			

Each F tests the simple effects of cancer_vs_ipsos_r within each level combination of the other effects shown. These tests are based on the linearly independent pairwise comparisons among the estimated marginal means.

Supplemental Table 9: Results of full models predicting COVID-specific variables

	Cancer Group					Comparison Group				
**Dependent Variable**	**Independent Variables**	**Beta**	**t**	**Sig.**	**Model F**	**Independent Variables**	**Beta**	**t**	**Sig.**	**Model F**
Risk/Hardship/ Conflict					34.22					52.10
	Age	-0.04	-1.04	.30		Age	-0.13	-3.35	**.00**	
	Difficulty Paying Bills	0.28	7.16	**.00**		Difficulty Paying Bills	0.36	9.81	**.00**	
	Education Level	-0.09	-2.57	**.01**		Education Level	0.08	2.29	**.02**	
	Gender	--	--	--		Gender	-0.06	-1.75	.08	
	Comorbidity Group	-0.02	-0.55	.58		Comorbidity Group	-0.04	-1.06	.29	
	QOL Composite	-0.20	-3.95	**.00**		QOL Composite	-0.12	-2.57	**.01**	
	Focused on Comparisons	0.10	2.72	**.01**		Focused on Comparisons	0.16	4.63	**.00**	
	Health Goals	--	--	--		Health Goals	0.15	4.42	**.00**	
	Negative Emphasis	0.14	3.66	**.00**		Negative Emphasis	0.19	5.04	**.00**	
	Positive Emphasis	--	--	--		Positive Emphasis	-0.01	-0.19	.85	
	Problem Goals	0.20	4.58	**.00**		Problem Goals	--	--	--	
	Recent Demands	0.16	4.28	**.00**		Recent Demands	0.17	5.30	**.00**	
	Negative Emphasis * QOL Composite	-0.13	-3.87	**.00**		Positive Emphasis* Comorbidity Group	0.12	2.16	**.03**	
	Problem Goals* QOL Composite	-0.08	-2.22	**.03**						
	Recent Demands * QOL Composite	-0.07	-1.98	**.05**						
Growth/ Support					42.38					42.30
	Difficulty Paying Bills	0.01	0.14	.89		Difficulty Paying Bills	--	--	--	
	Live with Spouse/Partner	0.13	3.78	**.00**		Live with Spouse/Partner	0.158	3.911	**.00**	
	Comorbidity Group	0.08	2.28	**.02**		Comorbidity Group	-0.003	-0.08	.94	
	QOL Composite	0.60	11.74	**.00**		QOL Composite	0.439	8.639	**.00**	
	Focused on Comparisons	0.16	4.60	**.00**		Focused on Comparisons	0.085	2.102	**.04**	
	Health Goals	0.19	5.29	**.00**		Health Goals	0.103	2.437	**.02**	
	Positive Emphasis	0.13	3.04	**.00**		Positive Emphasis	0.207	4.268	**.00**	
	Focused On Comparisons * QOL Composite	-0.11	-3.22	**.00**						
	Problem Goals* Comorbidity Group	0.08	2.14	**.03**						
Protect/Distress					36.99					14.23
	Age	--	--	--		Age	-0.06	-1.06	.29	
	Difficulty Paying Bills	0.20	4.58	**.00**		Difficulty Paying Bills	0.14	2.82	**.00**	
	Comorbidity Group	0.08	1.93	.05		Comorbidity Group	-0.03	-0.64	.52	
	QOL Composite	-0.25	-4.50	**.00**		QOL Composite	-0.21	-3.95	**.00**	
	Focused on Comparisons	--	--	--		Focused on Comparisons	0.20	4.18	**.00**	
	Health Goals	--	--	--		Health Goals	0.13	2.69	**.01**	
	Negative Emphasis	-0.10	-2.43	**.02**		Negative Emphasis	-0.14	-2.84	**.00**	
	Recent Demands	0.19	4.65	**.00**		Recent Demands	0.21	4.62	**.00**	
	Positive Emphasis * Comorbidity Group	-0.09	-2.11	**.04**		Recent Demands * QOL Composite	-0.15	-3.26	**.00**	
Altruism/ Coping					49.50					16.08
	Age	--	--	--		Age	0.02	0.48	.63	
	Difficulty Paying Bills	-0.18	-4.34	**.00**		Difficulty Paying Bills	--	--	--	
	Education Level	0.19	5.31	**.00**		Education Level	0.12	2.51	**.01**	
	Live with Spouse/Partner	0.10	2.72	**.01**		Live with Spouse/Partner	0.08	1.67	.10	
	Comorbidity Group	-0.01	-0.24	.81		Comorbidity Group	0.11	2.43	**.02**	
	QOL Composite	0.39	8.82	**.00**		QOL Composite	0.46	9.01	**.00**	
	Focused on Comparisons	--	--	--		Focused on Comparisons	0.13	2.87	**.00**	
	Negative Emphasis	-0.10	-2.55	**.01**		Negative Emphasis	0.11	2.26	**.02**	
						Recent Demands	0.01	0.20	.84	
						Recent Demands * Comorbidity Group	0.15	2.12	**.03**	

** All models were significant at p<0.001.*

Supplemental Table 10. Summary Schematic of COVID-Outcome Results by Group

	CANCER SURVIVORS							COMPARISON GROUP						
**APPRAISAL PROCESSES**	Focused on Comparisons	Problem Goals	Health Goals	Positive Emphasis	Negative Emphasis	Recent Demands		Focused on Comparisons	Problem Goals	Health Goals	Positive Emphasis	Negative Emphasis	Recent Demands	
Risk/Hardship/ Conflict	√ (+)	√ (+)			√ (+)	√ (+)		√ (+)		√ (+)		√ (+)	√ (+)	
Growth/Support	√ (+)		√ (+)	√ (+)				√ (+)		√ (+)	√ (+)			
Protect/Distress					√ (−)	√ (+)		√ (+)		√ (+)		√ (−)	√ (+)	
Altruism/Coping					√ (−)			√ (+)				√ (+)		
**DEMOGRAPHICS/PROs**	Age	Difficulty Paying Bills	Education Level	Gender	Live w Spouse / Partner	Comorbidity Group	QOL	Age	Difficulty Paying Bills	Education Level	Gender	Live w Spouse / Partner	Comorbidity Group	QOL
Risk/Hardship/Conflict		√ (+)	√ (−)				√ (−)	√ (−)	√ (+)	√ (+)				√ (−)
Growth/Support					√ (+)	√ (+)	√ (+)					√ (+)		√ (+)
Protect/Distress		√ (+)					√ (−)		√ (+)					√ (−)
Altruism/Coping		√ (−)	√ (+)		√ (+)		√ (+)			√ (+)			√ (+)	√ (+)

Legend: positive coefficient (green) *negative coefficient* (red)
